# Supplementary material for: Experimental reproducibility limits the correlation between mRNA and protein abundances in tumor proteomic profiles
Source: Cell Rep Methods. 2022 Sep 8;2(9):100288. doi: 10.1016/j.crmeth.2022.100288 (PMC9499981; doi:10.1016/j.crmeth.2022.100288)
Supplement: Document S1. Figures S1–S7 [file mmc1.pdf]

Cell Reports Methods, Volume *2*

Supplemental information

Experimental reproducibility limits the correlation between mRNA and protein abundances in tumor proteomic profiles

Swathi Ramachandra Upadhya and Colm J. Ryan

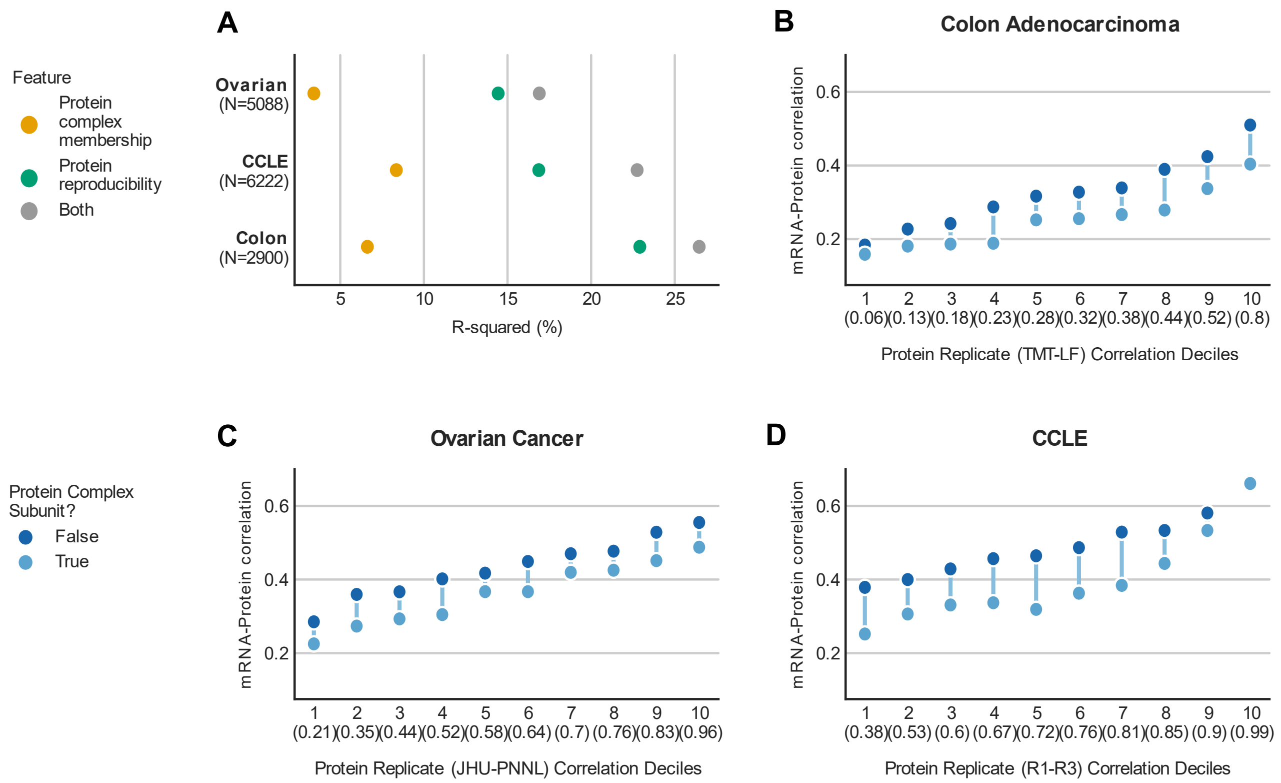

Figure S1. Protein complex membership and protein reproducibility contribute to the variation in mRNA-protein correlation. Related to Figure 2.

(A) Dot plot displaying the R-squared obtained from regressing mRNA-protein correlation of the indicated studies on protein complex membership and their corresponding protein reproducibility over the same set of proteins. The number of proteins considered for each analysis is specified in parentheses below the study on Y-axis. (B-D) Ranged dot plots showing the mean of mRNA-protein correlation for proteins that are complex subunits (light blue dot) or not (dark blue dot) within every decile of the proteomic replicates' correlation for colon (B) and ovarian tumour (C) and CCLE studies (D). The line represents the difference in the mean of the mRNA-protein correlation between the groups of proteins belonging to the same decile. X-axis indicates the decile number and contains the maximum correlation between the experimental proteomic replicates for that decile in parentheses.

A

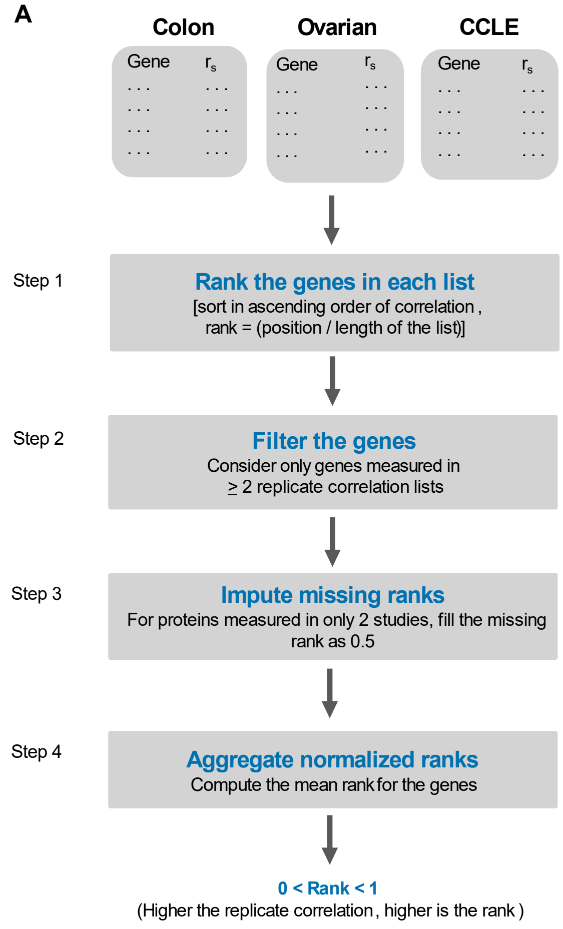

B

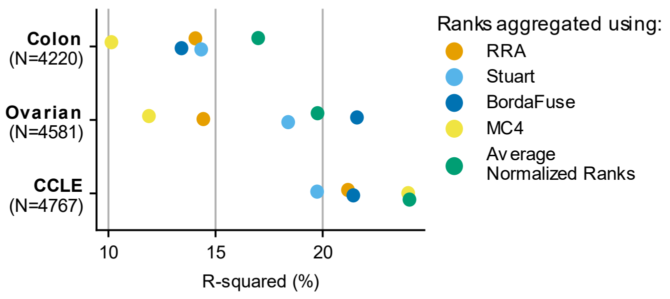

Figure S2. Aggregate protein reproducibility. Related to Figure 4.

(A) Workflow of our computational approach to aggregate the ranks of the correlation of experimental proteomic replicates from 3 different datasets - colon, ovarian and CCLE. The computed ranks lie between zero and one. The higher the correlation between the experimental proteomic replicates, the higher the rank. (B) Dot plot displaying R-squared obtained from regressing mRNA-protein correlation of the indicated studies on the aggregated protein ranks obtained from different algorithms (robust rank aggregation, Stuart, BordaFuse, Markov chain aggregator and our method of average normalized rank) over the same set of proteins. The number of proteins considered for each analysis is specified in parentheses below the study on Y-axis.

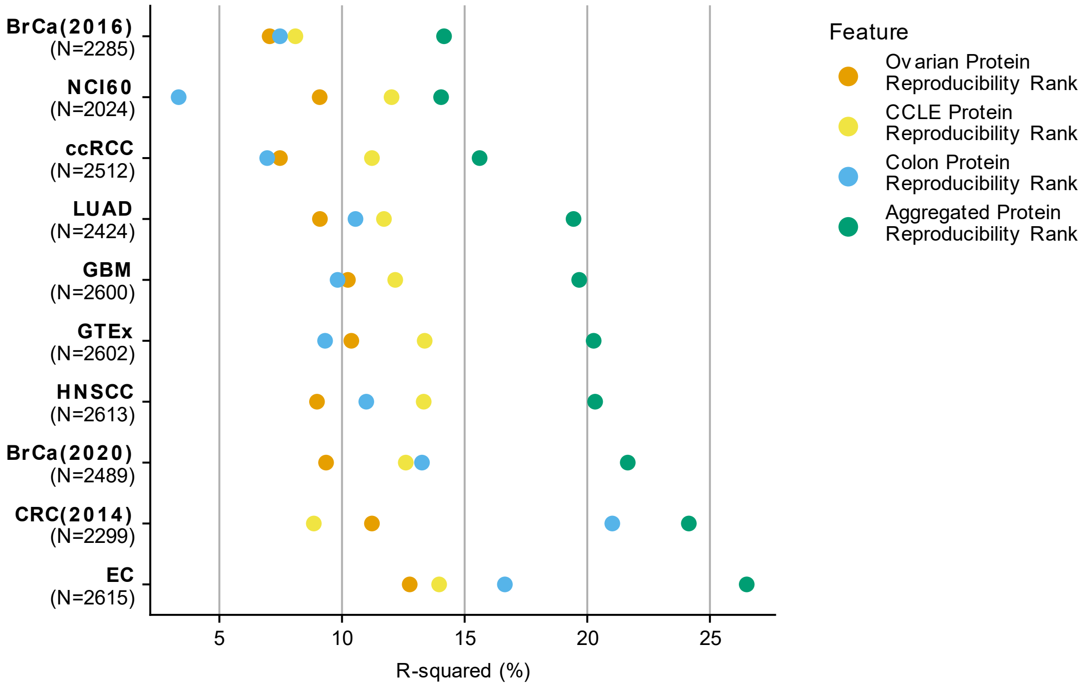

Figure S3. Aggregate protein reproducibility outperforms the individual protein reproducibility ranks in explaining the variation in mRNA-protein correlation. Related to Figure 4.

Dot plot comparing the R-squared values obtained from regressing mRNA-protein correlation on the individual protein reproducibility ranks and the aggregated protein reproducibility rank over the same set of proteins. The number of proteins considered for each analysis is specified in parentheses below the study on Y-axis.

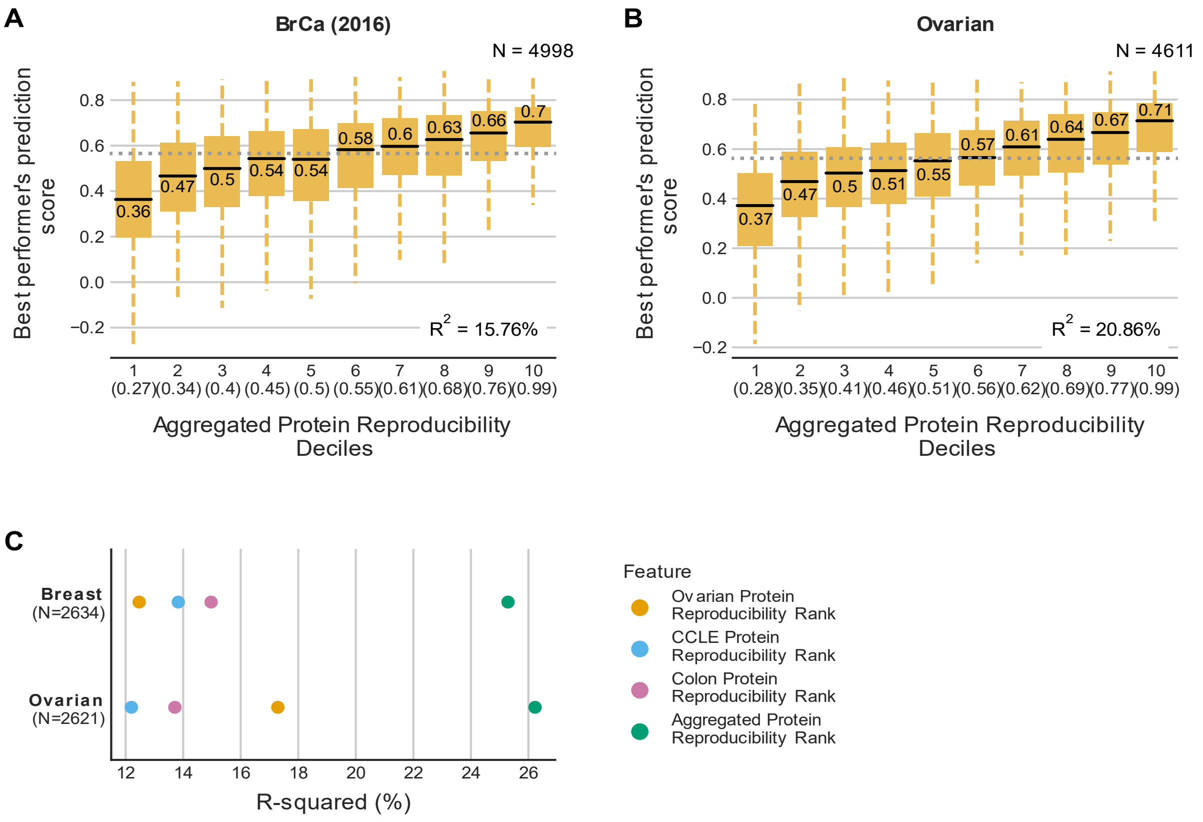

Figure S4. Proteins that are highly reproducible can be better predicted using machine learning. Related to Figure 4.

Boxplots showing the distribution of prediction scores from the best performing model in the NCI CPTAC DREAM Proteogenomics challenge for proteins in each decile of the aggregated protein reproducibility ranks in breast (A) and ovarian studies (B). The prediction score is the Pearson correlation between the observed and predicted protein abundance. The decile is indicated on the X-axis along with the highest score of the aggregated protein reproducibility rank present within that decile. For each box plot, the black central line represents the median, the top and bottom lines represent the 1st and 3rd quartile, and the whiskers extend to 1.5 times the interquartile range past the box. Outliers are not shown. The median of each decile is indicated above/below the black central line for each box plot. The overall median of the prediction score from the best performing model is indicated as a dotted gray line in each plot. The R^2 obtained from regressing the prediction score on the aggregated protein reproducibility ranks is in the bottom-right corner. (C) Dot plot comparing the R-squared values obtained from regressing protein abundance prediction scores of breast and ovarian tumour studies, obtained from NCI CPTAC Proteogenomics DREAM Challenge, on the individual protein reproducibility ranks and the aggregated protein reproducibility rank over the same set of proteins. The number of proteins considered for each analysis is specified in parentheses below the study on Y-axis.

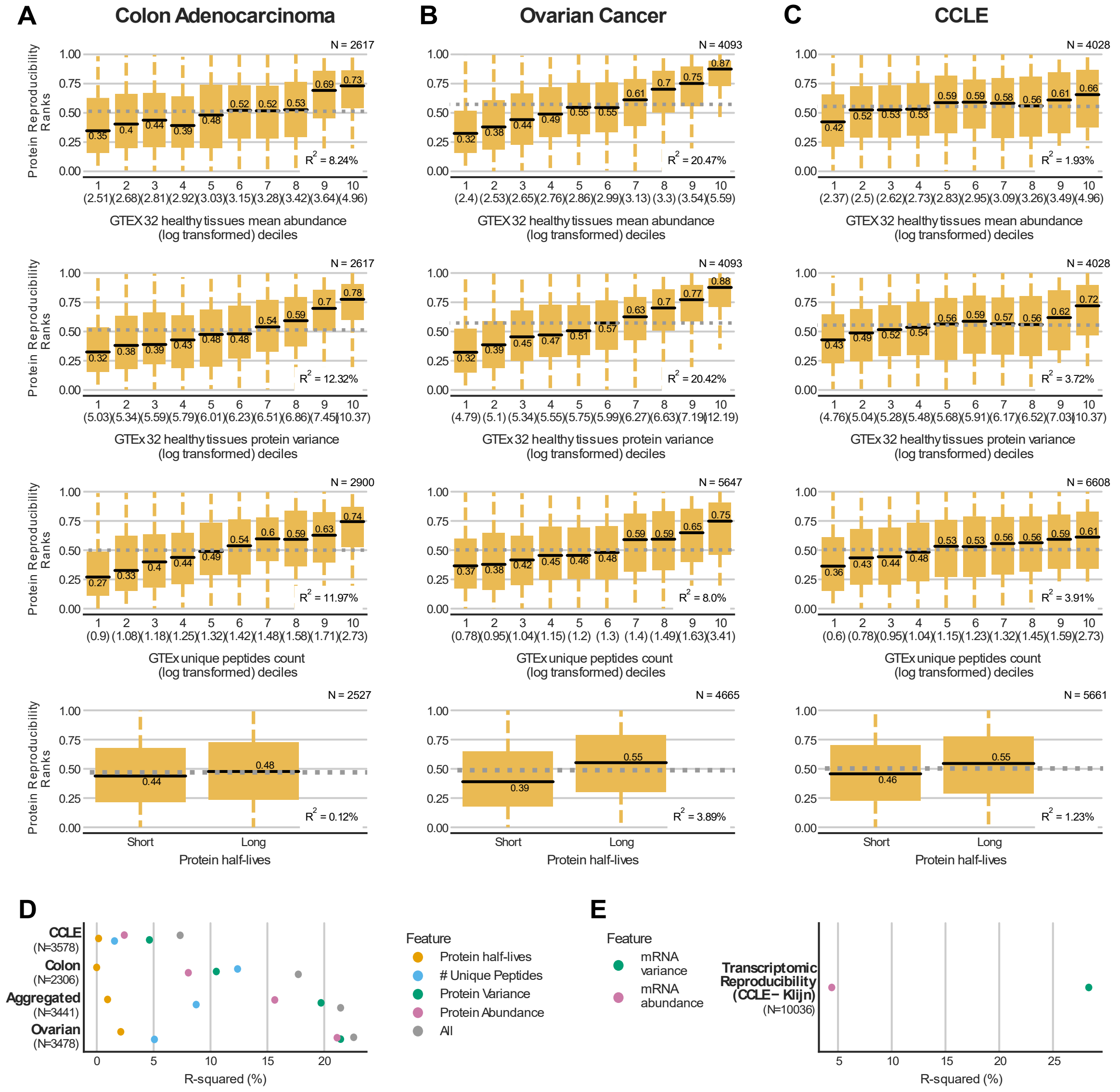

Figure S5. Potential factors influencing protein reproducibility (in individual studies with experimental proteomic replicates) and transcriptomic reproducibility. Related to Figure 5 and 6.

(A-C) Similar to Fig. 5 but for the individual protein reproducibility ranks from each study. (D) Dot plot comparing the R-squared values obtained from regressing the individual protein reproducibility ranks and the aggregated protein reproducibility ranks on the potential factors that affect protein reproducibility - protein abundance, variance of protein abundance, unique peptides, protein half-lives individually and all of them collectively over the same set of proteins. (E) Dot plot comparing the R-squared values obtained from regressing the potential factors that affect transcriptomic reproducibility – mRNA abundance and mRNA variance. The number of proteins considered for each analysis is specified in parentheses below the study on Y-axis.

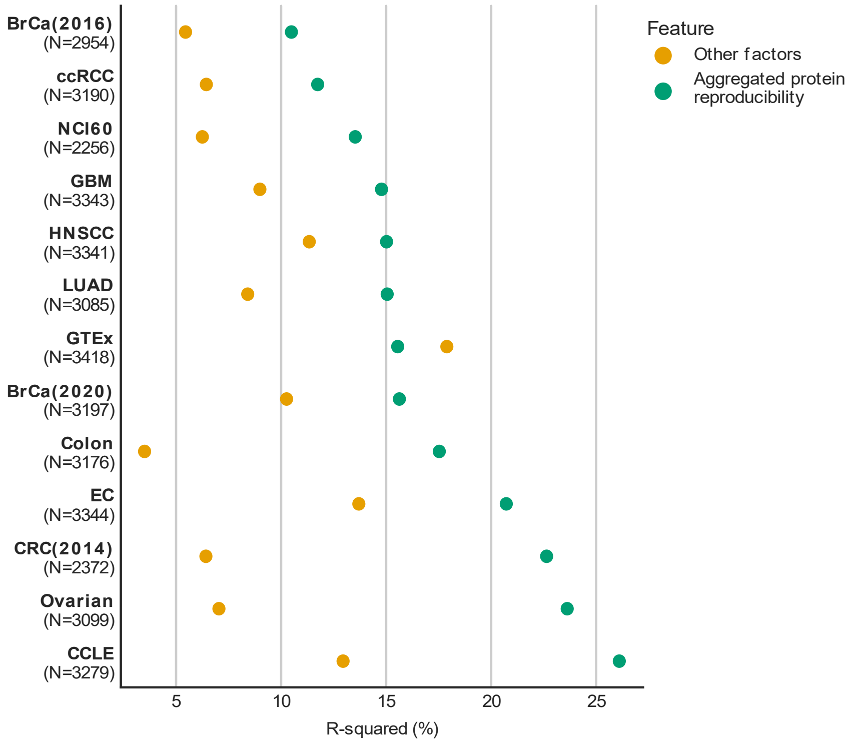

Figure S6. Protein reproducibility explains the variation in mRNA-protein correlation better than the other factors (protein abundance, protein variance, unique peptides, protein half-life). Related to Figure 5.
Dot plot comparing the R-squared values obtained from regressing mRNA-protein correlation of the studies on the aggregated protein reproducibility ranks and the potential factors that affect protein reproducibility - protein abundance, unique peptides, protein half-lives individually over the same set of proteins. The number of proteins considered for each analysis is specified in parentheses below the study on Y-axis.

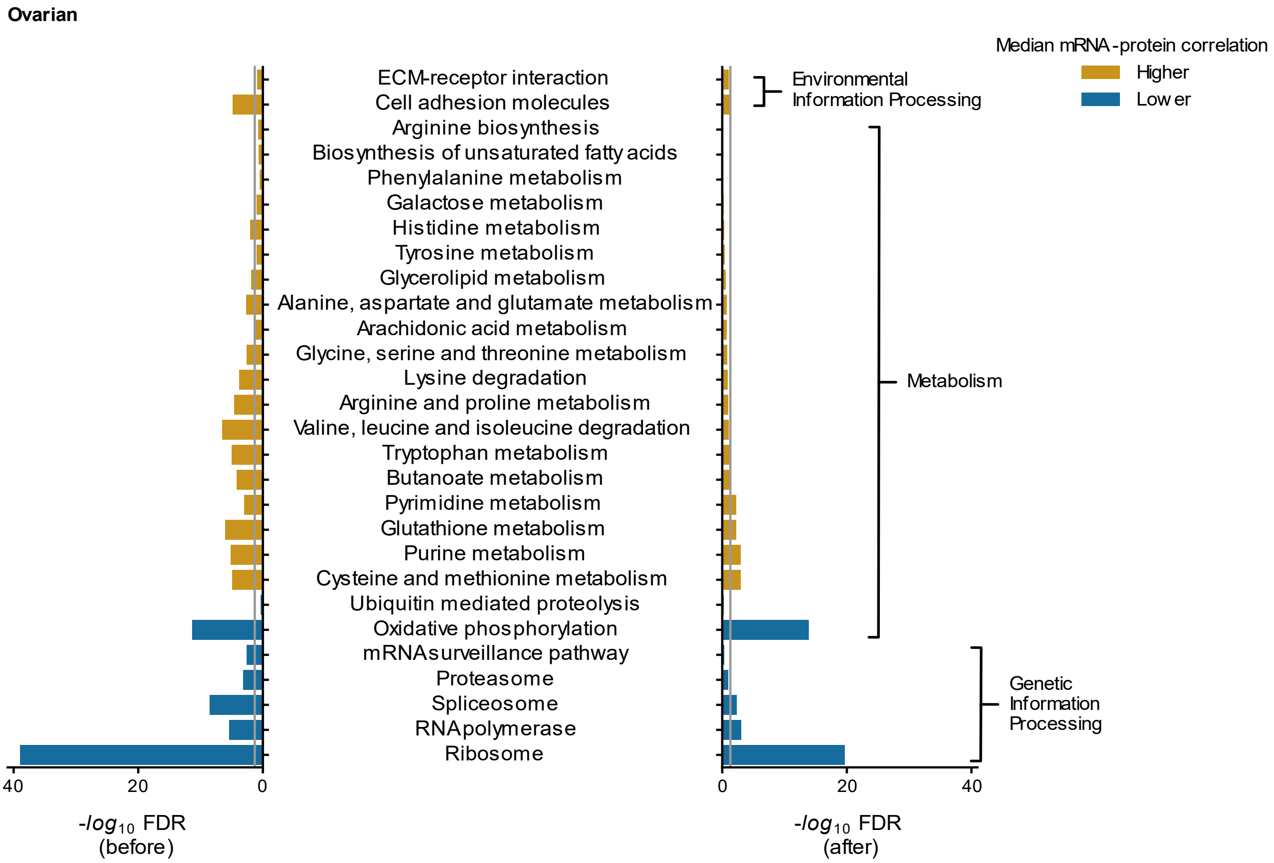

Figure S7. KEGG pathways enrichment analysis for ovarian cancer study. Related to Figure 7.
Bar charts displaying the KEGG pathway enrichment analysis of the ovarian cancer study mRNA-protein correlation before (left) and after (right) accounting for protein-protein and mRNA-mRNA reproducibility. The $-\log_{10}$ of Benjamini-Hochberg FDR corrected p-values calculated using Mann-Whitney U test is deemed as enrichment for the pathway. For each bar chart, the gray line indicates the threshold considered for significant enrichment (FDR < 0.05). If the enrichment is below the threshold, then it is not considered significant. The bars are coloured orange if the median mRNA-protein correlation of genes within the pathway > median mRNA-protein correlation of genes *not* in the pathway, otherwise the bars are coloured blue.
