# Supplementary material for: Experimental reproducibility limits the correlation between mRNA and protein abundances in tumor proteomic profiles
Source: Cell Rep Methods. 2022 Sep 8;2(9):100288. doi: 10.1016/j.crmeth.2022.100288 (PMC9499981; doi:10.1016/j.crmeth.2022.100288)
Supplement: Document S2. Article plus supplemental information [file mmc6.pdf]

Cell Reports Methods

Resource

Experimental reproducibility limits the correlation between mRNA and protein abundances in tumor proteomic profiles

Graphical abstract

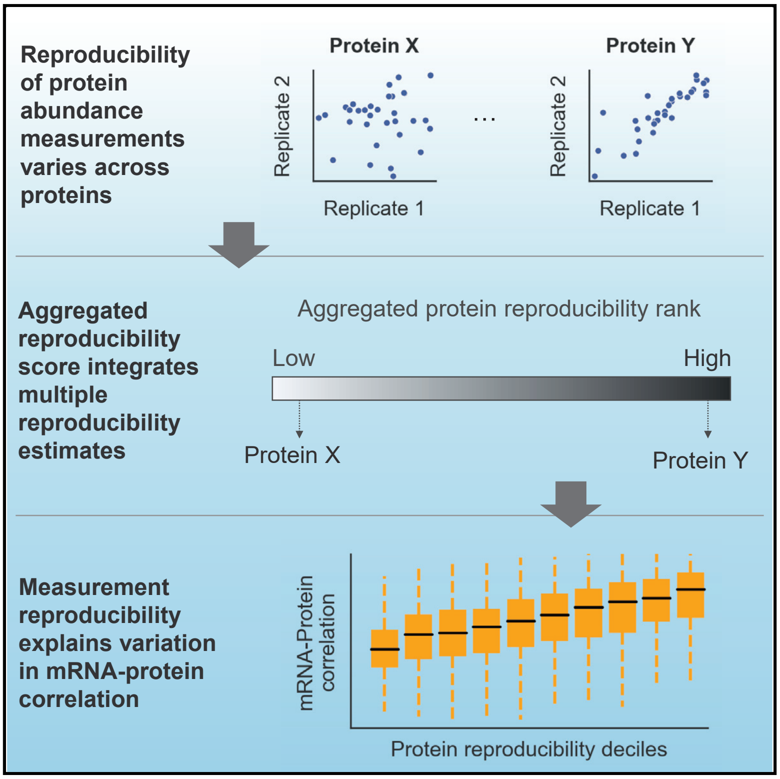

Authors

Swathi Ramachandra Upadhya, Colm J. Ryan

Correspondence

colm.ryan@ucd.ie

In brief

Upadhya and Ryan identified substantial variation in the reproducibility of protein abundance measurements across proteins. They develop an aggregate protein reproducibility score and show that this score explains variation in mRNA-protein correlations across multiple proteogenomic studies. They show that pathways with higher-than-average mRNA-protein correlations may simply be more reproducibly measured.

Highlights

- The reproducibility of protein abundance measurements varies across proteins
- Measurement reproducibility contributes to variation in mRNA-protein correlations
- Aggregated protein reproducibility score integrates multiple reproducibility estimates
- Pathways with high mRNA-protein correlations may just be more reproducibly measured

Upadhya & Ryan, 2022, Cell Reports Methods *2*, 100288
September 19, 2022 © 2022 The Authors.
https://doi.org/10.1016/j.crmeth.2022.100288

CellPress

Cell Reports Methods

CellPress
OPEN ACCESS

Resource

Experimental reproducibility limits the correlation between mRNA and protein abundances in tumor proteomic profiles

Swathi Ramachandra Upadhya[1,2] and Colm J. Ryan[1,2,3,*]
[1]School of Computer Science, University College Dublin, Dublin, Ireland
[2]Systems Biology Ireland, University College Dublin, Dublin, Ireland
[3]Lead contact
*Correspondence: colm.ryan@ucd.ie
https://doi.org/10.1016/j.crmeth.2022.100288

MOTIVATION Because they are easier to measure in high throughput, mRNA abundances are often used as a proxy measurement for protein abundances. However, there is only a moderate correlation between the two, and it is unclear to what extent this moderate correlation reflects post-transcriptional regulation and to what extent it can be attributed to measurement error. Here, by analyzing samples with replicate proteomes, we quantify the extent to which replicate measurements of the same proteins are correlated. We rank proteins according to their reproducibility and show that more reproducibly measured proteins have higher mRNA-protein correlation, suggesting that measurement error limits mRNA-protein correlation.

SUMMARY

Large-scale studies of human proteomes have revealed only a moderate correlation between mRNA and protein abundances. It is unclear to what extent this moderate correlation reflects post-transcriptional regulation and to what extent it reflects measurement error. Here, by analyzing replicate profiles of tumors and cell lines, we show that there is considerable variation in the reproducibility of measurements of transcripts and proteins from individual genes. Proteins with more reproducible measurements tend to have a higher mRNA-protein correlation, suggesting that measurement reproducibility accounts for a substantial fraction of the unexplained variation between mRNA and protein abundances. The reproducibility of individual proteins is somewhat consistent across studies, and we exploit this to develop an aggregate reproducibility score that explains a substantial amount of the variation in mRNA-protein correlations across multiple studies. Finally, we show that pathways previously reported to have a higher-than-average mRNA-protein correlation may simply contain members that can be more reproducibly quantified.

INTRODUCTION

Proteins are the primary actors in our cells, responsible for almost all biological activities. Therefore, understanding how protein abundances vary between healthy and disease states can provide an insight into how biological activities are altered in disease conditions. Among patients with the same disease, e.g., breast cancer, variation in protein abundances may explain differences in survival outcomes (Ősz et al., 2021) and drug responses (Shenoy et al., 2020). Consequently, significant efforts have been made recently to characterize proteomes across large patient cohorts (Ellis et al., 2013). However, our ability to quantify protein abundances at scale has lagged behind our ability to sequence genomes and quantify mRNA abundances. Large-scale efforts to molecularly characterize healthy and disease samples from humans have therefore primarily focused on DNA sequence variation and transcriptomic variation.

As transcriptomes are easier to quantify than proteomes, mRNA abundances are often used as a proxy for protein abundances. However, the relationship between mRNA abundances and protein abundances is complex and non-linear and varies significantly from protein to protein. Consistent with this, large-scale studies in humans and model organisms have revealed that for most genes there is only a moderate correlation between mRNA and protein abundances (Buccitelli and Selbach, 2020; Vogel and Marcotte, 2012). We note that correlations between mRNA and protein abundances can be calculated in two different ways: across all proteins within a given sample (i.e., in a given cell line, are the most abundant proteins also the most abundant transcripts?) or for a single protein across multiple

This is an open access article under the CC BY license (http://creativecommons.org/licenses/by/4.0/).

samples (i.e., do the samples with the highest levels of a specific protein also have the highest number of transcripts coding for that protein?) (Franks et al., 2017; Liu et al., 2016; Vogel and Marcotte, 2012). Here, we are concerned with variation across individuals, and so throughout when we discuss mRNA-protein correlations, we are calculating the correlation between the protein and transcript abundance for an individual protein across samples.

Tumor samples in particular have been subject to transcriptomic and proteomic profiling efforts, and these have provided insight into how variation in mRNA abundances across individuals is associated with variation in protein abundances across the same individuals. These studies have reported an average mRNA-protein correlation in the range of ∼0.2–0.5 (Mertins et al., 2016; Zhang et al., 2014, 2016). This moderate correlation between mRNA and protein abundances can be attributed to both biological and technical factors. Major biological factors that influence mRNA-protein correlation include translation rates that vary across proteins and conditions, highly variable half-lives for both proteins and mRNAs, and post-translational modifications that can alter protein stability and degradation (Buccitelli and Selbach, 2020).

Different proteins have been observed to have very different mRNA-protein correlations, and pathway enrichment analyses have identified specific functional groups with lower- or higher-than-average mRNA-protein correlations. For instance, a number of metabolic pathways have been shown to have higher-than average mRNA-protein correlations (Clark et al., 2019; Huang et al., 2021; Jarnuczak et al., 2021; Mertins et al., 2016; Zhang et al., 2014, 2016), suggesting limited post-transcriptional regulation of these proteins. In contrast, subunits of large protein complexes have been shown to have lower-than-average mRNA-protein correlations, suggesting significant post-transcriptional regulation (Gonçalves et al., 2017; Ryan et al., 2017; Taggart et al., 2020; Wang et al., 2017; Wu et al., 2013). Another factor that might influence mRNA-protein correlations across samples is the intrinsic variability in mRNA expression. mRNAs that do not vary across samples, such as those whose expression is usually tightly regulated, will not correlate with their corresponding proteins because variation is essential to observe correlation. As we focus our analysis on tumor profiles, where extensive copy-number alterations result in significant variation in mRNA abundances, this issue is a smaller concern.

Our technical ability to accurately and reproducibly quantify both mRNAs and proteins is potentially a major factor that influences the mRNA-protein correlation. If the error in our measurements is large, we would expect this error to reduce the correlation between mRNA and protein even in the absence of the biological factors outlined above. A number of studies have separately assessed the reproducibility of either mRNA ('t Hoen et al., 2013; Marioni et al., 2008; SEQC/MAQC-III Consortium, 2014) or proteomic (Casey et al., 2017; Tabb et al., 2010) profiling approaches. Others have explored how measurement errors in mRNA or proteomic profiling can influence the reported correlation between mRNA and protein abundances within sample correlations (across all proteins within a single sample/cell line) rather than across samples (for individual proteins across many samples) (Csárdi et al., 2015; Li et al., 2014).

Here, we analyze studies of tumors and cancer cell lines with replicate proteomic profiles in order to assess the impact of measurement reproducibility on mRNA-protein correlation that can be observed for individual proteins across samples.

RESULTS

A standardized pipeline reveals differences in the mRNA-protein correlation across studies

The average mRNA-protein correlation reported for different tumor proteomic profiling efforts varies substantially across studies—ranging from 0.23 in an early proteomic study of colorectal cancer (Zhang et al., 2014) to 0.53 in a recent study of lung adenocarcinoma (Gillette et al., 2020) (Table 1). However, it is not meaningful to directly compare the reported correlations because the methods used to quantify the mRNA-protein correlation have varied across studies—different studies have used different summary statistics (mean versus median), different correlation metrics (Pearson versus Spearman), and different criteria for protein inclusion (e.g., no missing values, at least 30% measured values, only the 10% most variable proteins) (Table 1). To enable a more direct comparison across studies, we calculated the mRNA-protein correlation for thirteen proteomic studies using a standardized pipeline. The datasets analyzed comprise ten studies of tumor samples (Clark et al., 2019; Dou et al., 2020; Gillette et al., 2020; Huang et al., 2021; Krug et al., 2020; Mertins et al., 2016; Vasaikar et al., 2019; Wang et al., 2021; Zhang et al., 2014, 2016), two studies of cancer cell lines (Guo et al., 2019; Nusinow et al., 2020), and one study of healthy tissues (Jiang et al., 2020). Within each study, we calculated the median Spearman correlation between mRNA and protein for all proteins that were measured in at least 80% of samples (STAR Methods; Tables 1 and S1). Applying the same pipeline using Pearson correlation rather than Spearman correlation revealed broadly similar results (Table 1), and so throughout the remainder of the paper, we focus our analysis on correlation calculated using Spearman correlation as it is the metric most commonly used in proteogenomic studies (9 of 13 studies).

Across all studies, the median recalculated correlation was 0.43 with a maximum of 0.55 (lung adenocarcinoma [LUAD]; Gillette et al., 2020) and a minimum of 0.21 (colorectal cancer [CRC]; Zhang et al., 2014). In some instances, the recalculated correlation was similar to that originally reported, but in others there was a substantial difference. For example, the correlation recalculated for endometrial cancer (0.48) was the same as originally reported (Dou et al., 2020), while the recalculated correlation for colon cancer was much lower than that reported by the authors (0.27 versus 0.48) (Vasaikar et al., 2019). This is because the colon cancer study reported the mean mRNA-protein correlation for only the 10% most variable proteins rather than the full set of proteins. These highly variable proteins have higher than average mRNA-protein correlations.

More recent studies appear to have higher mRNA-protein correlations, e.g., we observe a mean of 0.49 for studies published after 2019 versus 0.35 for studies published in 2016 or earlier (Table 1). This cannot simply be attributed to differences in the cancer types studied in different years, as the two cancer types profiled twice (colon and breast) see an improvement from the

Table 1. Analysis of mRNA-protein correlation using a standardized pipeline

Data	Published year	Reported correlation	Protein inclusion criterion in reported correlation	Computed median Spearman correlation	Computed median Pearson correlation
GTEx 32 healthy tissues (GTEx)	2020	0.46	<5 tissues with missing values for both protein and RNA measurements	0.51	0.59
Cancer Cell Line Encyclopaedia (CCLE)	2020	0.48	quantified in at least one ten-plex (9 cell lines)	0.46	0.48
NCI-60 cancer cell lines (NCI60)	2019	not reported	–	0.36	0.40
Glioblastoma (GBM)	2021	not reported	–	0.50	0.51
Head and neck squamous cell carcinoma (HNSCC)	2021	0.52	<50% missing values	0.54	0.56
Lung adenocarcinoma (LUAD)	2020	0.53	<50% missing values	0.55	0.56
Endometrial cancer (EC)	2020	0.48	contain mRNA and protein measurements across all patients	0.48	0.51
Breast cancer (BrCa 2020)	2020	0.41	contain mRNA and protein measurements (proteins <70% missing values)	0.44	0.43
Clear cell renal carcinoma (ccRCC)	2019	0.43	contain mRNA and protein measurements across all patients	0.41	0.42
Colon cancer (colon)	2019	0.48	top 10% most variably expressed proteins quantified in both platforms	0.27	0.28
Ovarian cancer (ovarian)	2016	0.45	contain mRNA and protein measurements across all patients	0.41	0.41
Breast cancer (BrCa 2016)	2016	0.39	contain mRNA and protein measurements across all patients passing quality control checks.	0.42	0.42
Colon and rectal cancer (CRC 2014)	2014	0.23	protein measurement with average spectral count across all patients ≥ 1.4	0.21	0.22

earlier studies (Table 1). This would suggest that technical and experimental factors may influence the reported mRNA-protein correlations and that improvements in either technology or experimental protocols have resulted in improved mRNA-protein correlations over time.

The correlation across replicate proteomic profiles is only moderate

To assess the reproducibility of mass spectrometry-based proteomic measurements, we analyzed three studies containing replicate proteomic profiles: ovarian tumor samples (Zhang et al., 2016), colon tumor samples (Vasaikar et al., 2019), and cancer cell lines of mixed lineages from the Cancer Cell Line Encyclopedia (CCLE) (Nusinow et al., 2020) (Figure 1A). The nature of the replicates varies across the different studies: for ovarian cancer, the same tumor sample was profiled in two different laboratories, for the cancer cell lines, biological replicates were performed within the same lab 1 year apart, while for colon cancer, the same tumor samples were profiled with two different mass spectrometry (MS) techniques, i.e., isotope-based protein quantification (TMT-10) and label-free spectral counting MS. Thus, there is diversity in the replicate proteomic profiles in terms of sample types (tumor samples and cancer cell lines), sites, and techniques used to quantify the proteins.

Many biological factors that influence mRNA-protein correlation, such as post-transcriptional regulation, are not relevant in the case of replicate measurements of proteins, and so we expected the replicate proteomic profiles to be more highly correlated than mRNA and protein profiles. This was indeed the case for all studies. The median protein-protein reproducibility for the replicate proteomic profiles from the CCLE dataset was 0.72 (Figure 1B; Table S2), whereas the median mRNA-protein correlation was only 0.48 (Table 1). The median protein-protein reproducibility for the replicate proteomic profiles of ovarian tumors was 0.57 (Figure 1B), which is higher than the median mRNA-protein correlation of 0.41 (Table 1). The replicate protein-protein reproducibility for the colon study (median 0.28) was much lower than that observed for the other studies. However, it was still higher than the median-calculated mRNA-protein correlation (0.21). One reason for the colon study to have a low median protein-protein reproducibility is that one of the two replicate proteomic profiles is quantified using label-free/spectral counting MS, which is not as accurate as the stable isotope-based protein quantification methods (Liu et al., 2016). Overall, we can conclude that although protein-protein reproducibility is consistently higher than mRNA-protein correlations, the protein-protein reproducibility is still only moderate.

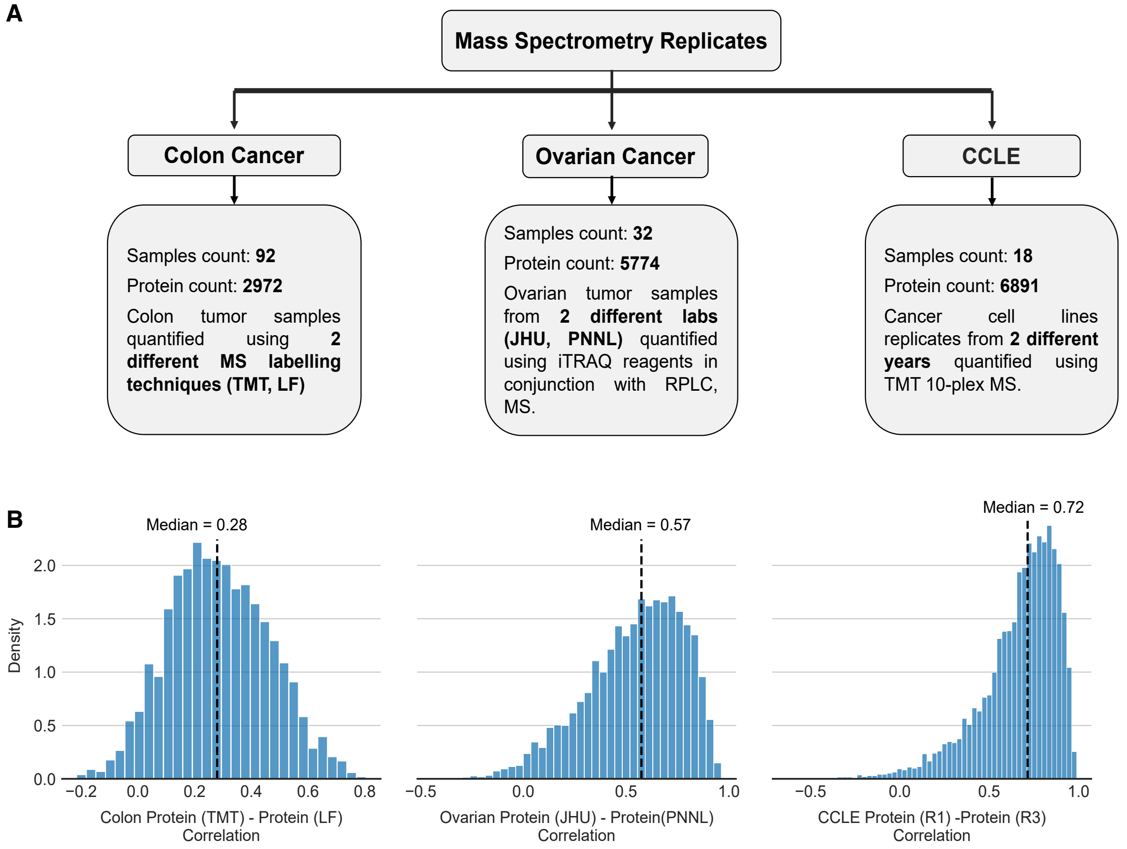

Figure 1. Protein-protein reproducibility across replicates is moderate and variable

(A) Overview of the replicates available for the three different proteomic studies.

(B) For each study, we calculate the Spearman correlation for individual proteins across the proteomic replicates. The distribution of the protein-protein reproducibility is shown in the histogram for all measured proteins. For each study, the black dashed line represents the median.

Proteins with higher reproducibility have higher mRNA-protein correlation

The moderate correlations reported between mRNA and protein abundances have been attributed to a variety of biological factors, including post-transcriptional regulation, varying translation rates, and varying degradation rates (Buccitelli and Selbach, 2020; Payne, 2015; Vogel and Marcotte, 2012). However, our observation that some proteins can be quantified more reproducibly than others suggests that noise in quantification may also be a major factor. If this is the case, we would expect that proteins that can be more reproducibly quantified will have a higher mRNA-protein correlation. To assess this, for each study we used the replicate proteomic profiles to stratify the proteins into deciles, ranging from the 10% of proteins with the lowest protein-protein reproducibility to the 10% with the highest protein-protein reproducibility (STAR Methods). We then calculated the mRNA-protein correlation for all of the proteins within each decile. We found, for all three studies, that the median mRNA-protein correlation increases with protein-protein reproducibility (Figure 2). The colon cancer study shows a difference in the median mRNA-protein correlation of 0.33 between the first and last deciles of protein reproducibility. Similarly, ovarian cancer data show a difference of 0.35, and the CCLE data show a difference of 0.37. This indicates that the reproducibility of proteomic measurements has a major impact on the calculated mRNA-protein correlation. We used a linear regression model to understand how much of the variation in mRNA-protein correlation can be explained by variation in protein-protein reproducibility and found that it explains approximately 14%, 17%, and 23% in the ovarian, CCLE, and colon studies, respectively (STAR Methods; Figure 2 and S1A).

Previous work has identified protein complex membership as the factor most predictive of variation in mRNA-protein correlation, with subunits of protein complexes typically having lower-than-average mRNA-protein correlation (Gonçalves et al., 2017; Ryan et al., 2017). Using the same linear modeling approach as above, we found that protein complex membership explains approximately 3%, 8%, and 6.7% of the variation in the ovarian, CCLE, and colon studies, respectively (Figure S1A). This suggests that noise in the quantification of protein abundances explains much more (on average ~3 times) of the variance in mRNA-protein correlation than the most predictive previously identified factor. Combined, the protein-protein reproducibility and protein complex membership features explained approximately 17%, 23%, and 26% of the variation in mRNA-protein correlation in the ovarian, CCLE, and colon studies, respectively

CellPress
OPEN ACCESS

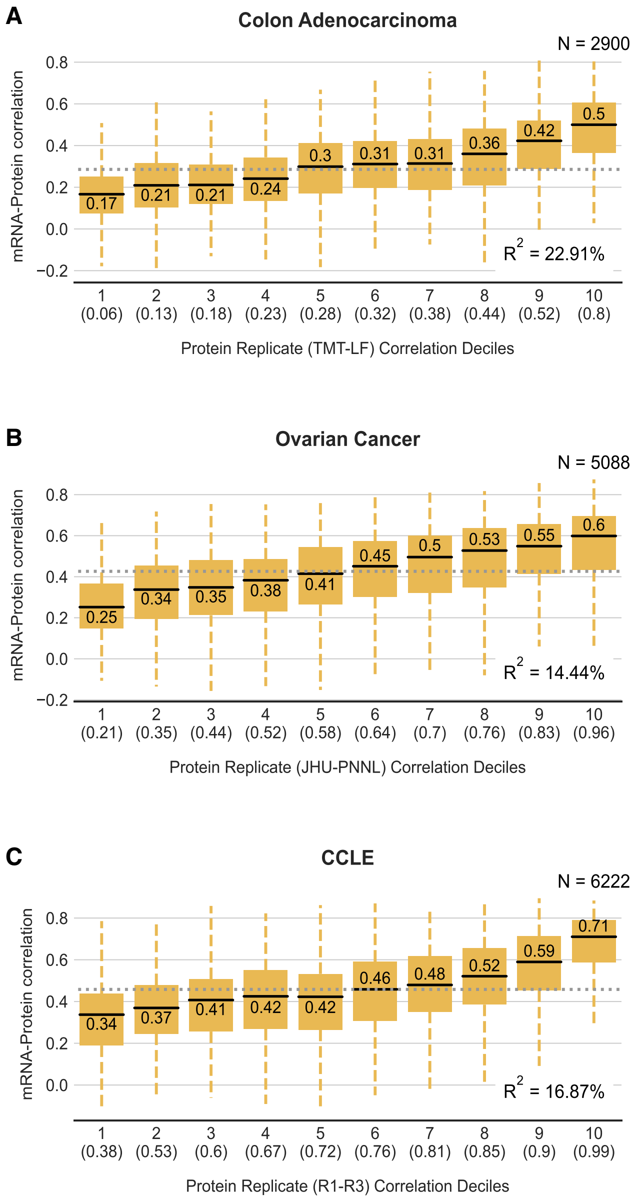

Figure 2. Proteins with higher reproducibility have higher mRNA-protein correlation

(A–C) Boxplots showing the distribution of mRNA-protein correlation for proteins binned according to their protein-protein reproducibility in the colon (A), ovarian (B), and CCLE (C) studies. The total number of proteins considered for each plot is indicated at the top right corner. The bins are deciles—each containing ~10% of the proteins. The decile is indicated on the x axis along with the highest correlation between experimental replicates present within that decile. For each box plot, the black central line represents the median, the top and bottom lines represent the 1st and 3rd quartiles, and the whiskers extend to 1.5 times the interquartile range past the box. Outliers are not shown. The median of each decile is indicated above/below the black central line for each box plot. The median mRNA-protein correlation across all proteins for each study is indicated as a dotted gray line in each plot. The R^2 obtained from regressing the mRNA-protein correlation on protein-protein reproducibility is in the bottom right corner.

(Figure S1A). This is significantly more than protein complex membership or protein-protein reproducibility alone ($p < 0.001$, likelihood ratio test), suggesting that protein complex membership and protein reproducibility independently contribute to the variation in mRNA-protein correlation. This is also evident when binning proteins into reproducibility deciles—although proteins that are complex subunits are present in every decile, they have consistently lower mRNA-protein correlations (Figures S1B–S1D).

Proteins with high reproducibility in one study are also highly reproducible in other studies

In addition to providing a summary of how reproducible the protein measurements from each study are on average, the replicate profiles enable us to see which proteins are most reproducibly quantified overall. In the CCLE study, the median correlation between replicate measurements calculated across all proteins was 0.72, but this ranged from −0.2 to 1.0 for individual proteins. Similarly, the median for all proteins in the ovarian study was 0.57, but the individual correlations ranged from −0.6 to 1.0, and the median for the colon tumor study was 0.28 with a range from −0.2 to 0.8. This suggests that, at least within individual studies, some proteins may be more reproducibly quantified than others.

To understand whether the same proteins were reproducibly quantified across multiple studies, we analyzed pairs of studies together. We found that there was a moderate correlation (0.38) between the protein reproducibility calculated using the ovarian tumor replicates and the colon cancer replicates (Figure 3A). Combinations of other pairs of studies revealed similar moderate correlations: colon and CCLE (0.31) and ovarian and CCLE (0.24) (Figures 3B and 3C). Although the nature of the samples (tumor versus cell line) and the quantification approaches (TMT/label-free quantification) varied across studies, this suggests that there is some agreement in terms of which proteins can be reproducibly quantified. In general, proteins that are highly reproducible in one study tend to be highly reproducible in others, while proteins that show poor reproducibility in one study tend to show poor reproducibility in others (Figure 3). For example, GBP1 is one of the proteins with reproducibility that is consistently high across all three studies (Figure 3D), while RPS29 has consistent low reproducibility (Figure 3E).

An integrated ranking of protein reproducibility partially explains the variable mRNA-protein correlation in 10 additional studies

Proteogenomic studies with large numbers of replicates, such as the three we analyzed above, are the exception rather than the rule. Consequently, for most studies, we do not know how reproducible the proteomic measurements are. However, as noted above, proteins that are highly reproducibly quantified in one study are more likely to be highly reproducible in others. We therefore sought to aggregate the replicate protein correlations from all three studies (CCLE, ovarian, colon) into a single list containing a ranking of protein reproducibility (STAR Methods; Figure S2A; Table S2). We evaluated a number of different aggregation approaches and found that a simple method using average normalized rank explained the most variance in mRNA-protein

correlations of the three studies containing proteomic replicates (STAR Methods; Figure S2B). We used this approach to create a ranked order of protein reproducibility for the 5,211 proteins that were quantified in at least two out of the three studies. We then used this aggregated list to assess the extent to which "average" protein reproducibility explains the varying mRNA-protein correlations observed in ten other studies (Clark et al., 2019; Dou et al., 2020; Gillette et al., 2020; Guo et al., 2019; Huang et al., 2021; Jiang et al., 2020; Krug et al., 2020; Mertins et al., 2016; Wang et al., 2021; Zhang et al., 2014) (Figure 4). For all these studies, we find that proteins with more reproducible measurements tend to have higher mRNA-protein correlations. Although the aggregated ranks are based on data from cancer studies, we observe the same trend in healthy tissues obtained from the GTEx project (Figure 4J). Similarly, although the aggregated ranks are generated using studies that quantify proteins through data-dependent acquisition (DDA) approaches, we observed the same trend for a study that quantified proteins using data-independent acquisition (DIA)-based proteomics (sequential window acquisition of all theoretical mass spectra [SWATH-MS]) in the NCI-60 cancer cell lines (Figure 4I). In general, the mRNA-protein correlation increases with protein reproducibility for samples from both healthy and diseased conditions and irrespective of the proteomic quantification approach.

To quantify the amount of variation in mRNA-protein correlation that could be explained by our aggregated protein reproducibility ranks, we used a linear regression model for the ten different studies. We found that the aggregated ranks explain ~10%–20% (median 14%) of the variation in these studies (Figure 4).

To test if there was an advantage to using the aggregate protein reproducibility over protein reproducibility measured in either of the three individual studies (CCLE, ovarian, colon), we compared the variance explained by the aggregate ranks with that explained by each individual study. In all ten studies without proteomic replicates, the aggregated ranks explained the variation in mRNA-protein correlation better than the ranks from any individual dataset (Figure S3).

A number of efforts have been made to use machine learning to predict protein abundances from mRNA abundances (Fortelny et al., 2017; Li et al., 2019; Yang et al., 2020). Recently, the NCI-CPTAC DREAM proteogenomics challenge engaged the community to predict protein abundances of breast and ovarian tumor profiles using their corresponding genomic and transcriptomic information (Yang et al., 2020). We hypothesized that proteins whose measurements are highly reproducible could be predicted better using machine-learning algorithms. Hence, we analyzed the prediction scores from the best-performing model using the protein reproducibility data. We observed a stark difference in the prediction scores of the lowest and highest deciles of the protein reproducibility (Figures S4A and S4B). While the lowest decile has a correlation of ~0.35 between the measurements and predictions, the highest decile has a correlation of ~0.7. The aggregated protein reproducibility ranks could explain ~25% and 26% of the variation in the prediction scores of breast and ovarian cancer studies, respectively, again outperforming the reproducibility measured in any individual study (Figure S4C).

Protein measurement reproducibility is influenced by abundance, variance, and unique peptides

To understand what causes some proteins to be more reproducibly measured than others, we analyzed a number of factors that we hypothesized might influence the reliability of their measurements.

All of the studies analyzed here make use of "bottom-up" quantification approaches where proteins are first digested into peptides; these peptides are then quantified using a mass spectrometer, and peptide quantifications are converted into protein abundances computationally. This quantification is a stochastic process, and there is no guarantee that every peptide in a given sample will be detected by the mass spectrometer. The quantification of proteins that have low abundance, and hence fewer detectable peptides, is especially likely to be subject to substantial stochastic variation. A small number of peptides missed can make a big difference to the quantification of these low abundance proteins, while for highly abundant proteins, a few extra or missing peptides will make only a small difference. To assess the contribution of protein abundance to protein measurement reproducibility, we obtained the protein abundances measured in 201 tissue samples from 32 healthy human tissues collected by the GTEx project (Jiang et al., 2020). For each protein, we calculated the mean abundance across all samples and tissues. We found a clear relationship between the mean protein abundance and the aggregated protein reproducibility rank—more abundant proteins are more reproducibly measured (Figure 5A). We performed a similar analysis for the three individual proteomic replicate studies and found the result to be consistent (Figures S5A–S5C).

Proteins whose abundances do not vary significantly across individuals are unlikely to have high mRNA-protein correlations, as correlation measures are dependent on there being meaningful variation in the data. Furthermore, as the variation observed experimentally is likely a combination of both real biological variation and experimental noise, proteins with lower biological variation in abundance will tend to be more affected by measurement noise. For each protein, we computed the variance in protein abundance across samples from the GTEx project (Jiang et al., 2020). We then assessed the influence of this variance on the reproducibility of measurements of individual proteins. Similar to the mean protein abundance above, we found that proteins with a higher variance of protein abundance are more reproducibly measured (Figure 5B). Furthermore, the variance of protein abundance explains ~20% of the variation in the aggregated protein reproducibility ranks. Similar trends were observed for the three individual proteomic replicate studies (Figures S5A–S5C).

The number of unique peptides generated per protein is also crucial for protein quantification by MS. To assess the impact of this, we identified the number of unique peptides identified per protein using the GTEx study. We stratified all proteins into deciles based on the number of unique peptides identified and found that the aggregated protein reproducibility increased with every decile of unique peptides identified (Figure 5C). This pattern was also evident in the protein reproducibility measured in each of the three individual studies (Figures S5A–S5C). Thus, the more unique peptides identified per protein, the higher the confidence of the measured protein levels.

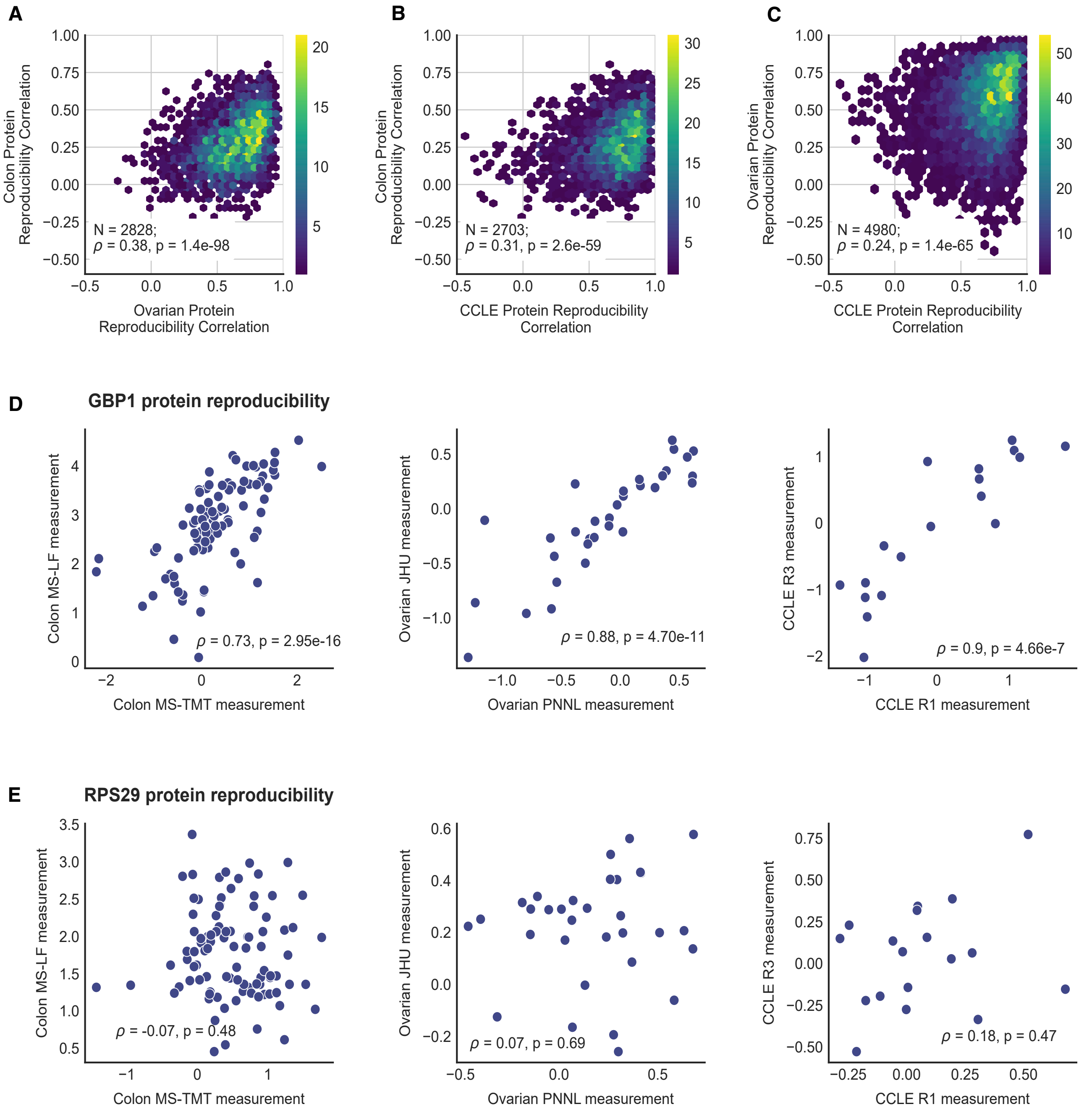

Figure 3. Proteins with high reproducibility in one study are also highly reproducible in other studies

(A–C) Binned heatmaps showing the relationship between the protein-protein reproducibility calculated in different studies. Each heatmap shows the relationship between two studies, indicated on the x and y axes. The regions of the heatmaps are colored according to the number of proteins present in the region as indicated in the color bar. The number of proteins in common and Spearman correlation between the two studies, with the associated p value, are specified in the box for each of the plots.

(D and E) For each study with experimental protein replicates, scatter plots illustrating the relationship between protein-protein reproducibility are shown for a protein with high reproducibility, GBP1 (D), and a protein with low reproducibility, RPS29 (E). For each scatter plot, the Spearman correlation coefficient of the protein-protein reproducibility and the associated p value is indicated at the bottom.

One of the biological reasons proposed for the weak mRNA-protein correlation is the difference in mRNA and protein half-lives (Vogel and Marcotte, 2012). mRNAs typically have a half-life of 2.6–7 h, while proteins have half-lives ranging from a few seconds to a few days (Vogel and Marcotte, 2012). Recently, proteins with longer half-lives were found to be more predictable using machine

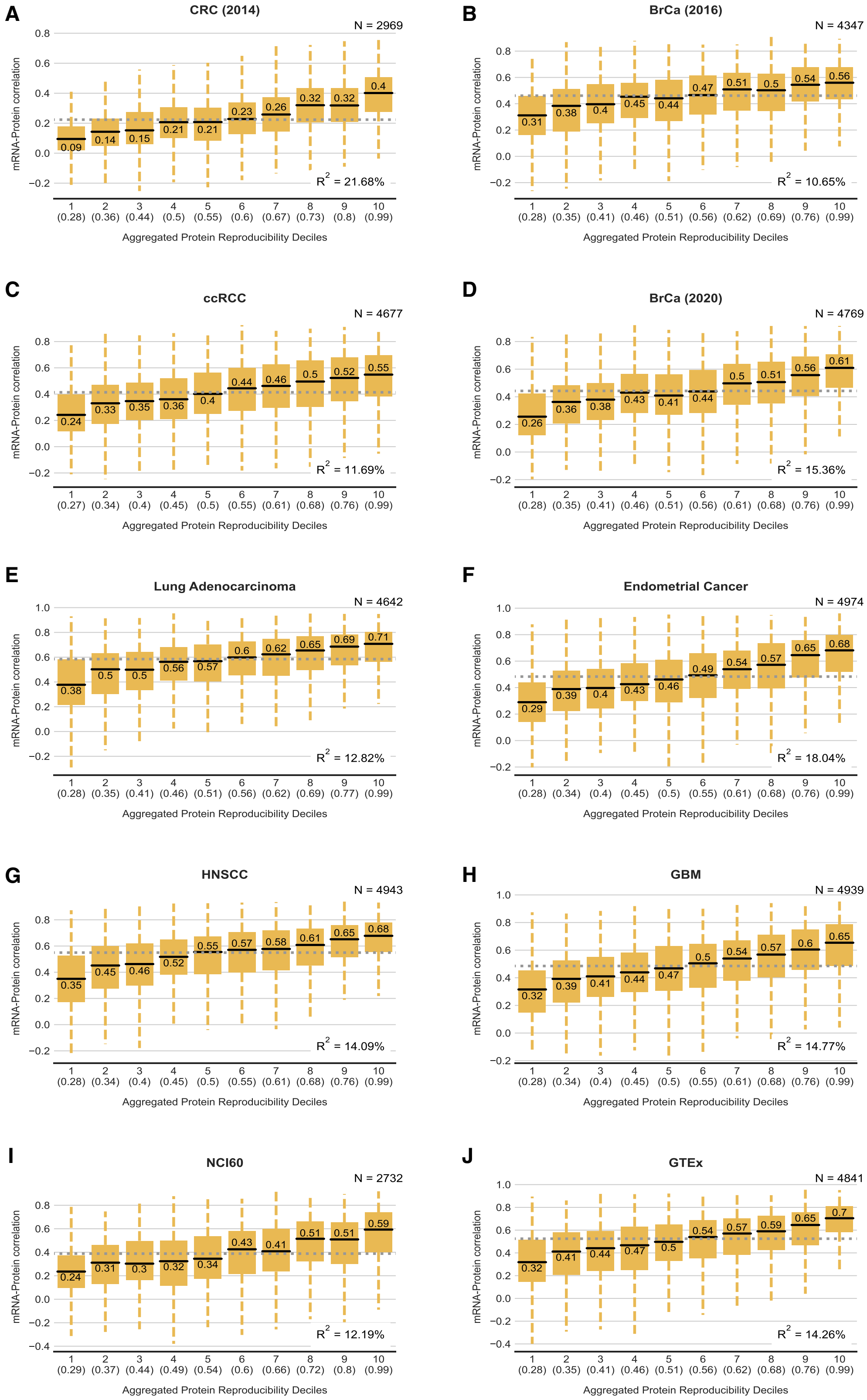

(legend on next page)

learning, irrespective of the transcript half-lives (Yang et al., 2020). This led us to assess protein half-life as a potential factor for the reproducibility of protein measurements. We obtained protein half-lives estimations from a previous publication (Zecha et al., 2018) and divided them into two categories—long and short half-lives (STAR Methods)—as was done in Yang et al. (2020). Although both categories contain proteins with reproducibility scores ranging from 0 to 1, proteins with a long half-life have a higher median protein reproducibility score ($p = 9.70e-25$, Mann-Whitney U test, two-sided; Figures 5D and S5A–S5C).

We note that there is some correlation between the attributes considered, in particular more abundant proteins tend to have more unique peptides identified. To understand the relative contribution of each factor, we performed rank regression by using the individual factors as the explanatory variables and the ranks of the proteomic reproducibility as the response variable (STAR Methods). We found in all cases that a model including all four factors performed better than a model including only the best individual factor, suggesting that variance in reproducibility can best be explained by a combination of factors (Figure S5D).

The factors above all contribute to protein-protein reproducibility, raising the question of whether they themselves might be sufficient to explain variation in mRNA-protein correlation. To assess this, we performed linear regression with these factors (abundance, variance, unique peptides, and protein half-lives) as explanatory variables and the mRNA-protein correlation of each of the 13 different studies as response variables. We found that a combined model of the factors explained ~3%–17% of the variation in mRNA-protein correlation of the different studies (Figure S6). However, the aggregated protein reproducibility explains a considerably higher percentage of the variation in mRNA-protein correlation in 12 of 13 studies. The GTEx study is the lone exception, likely a result of the independent variables (protein abundance, variance, number of unique peptides) being calculated from the GTEx study itself (Figure S6).

Transcriptomic reproducibility also contributes to the variance in mRNA-protein correlation

Thus far, we have primarily focused on understanding the influence of protein quantification reproducibility on mRNA-protein correlation. However, it is also likely that the reproducibility of mRNA measurements is an important factor in determining mRNA-protein correlations. To assess the impact of transcriptomic reproducibility on mRNA-protein correlation, we compared transcriptomic profiles for 382 cancer cell lines from the CCLE (Ghandi et al., 2019) with those generated in a separate profiling effort (Klijn et al., 2015). We find that the median gene-wise Spearman correlation across studies was 0.75 (STAR Methods; Figure 6A). Again, this varied significantly across transcripts, ranging from −0.05 to 0.96. As with protein reproducibility, we find that transcriptomic reproducibility is influenced by both mRNA abundance and variance (STAR Methods; Figure S5E).

We used a linear regression model to quantify, in all thirteen proteogenomic studies, how much of the variation in mRNA-protein correlation could be explained by transcriptomic reproducibility. We found that the median variance explained was 15%. In most studies (8/13), our aggregated protein reproducibility explained a higher proportion of the variance than the mRNA reproducibility (Figure 6B).

Compared with the other studies, the CCLE study had a strikingly higher percentage of variance explained by transcriptomic reproducibility (40%). This is presumably because there is a large overlap in the set of samples used to compute the transcriptomic reproducibility and the CCLE mRNA-protein correlation, unlike the other studies. For the CCLE, the variance explained by mRNA-mRNA reproducibility is higher than the variance explained by protein-protein reproducibility. However, the mRNA-mRNA reproducibility was estimated using a much higher number of cell lines (382 versus 18 for protein-protein reproducibility), which we reasoned could explain the increased variance explained. To test this hypothesis, we downsampled the available transcriptomic data to make the comparison more equal (sampling 18 cell lines with transcriptomes at random; STAR Methods). We found that, using this approach, the protein-protein reproducibility explained more of the mRNA-protein variability than the mRNA-mRNA reproducibility (on average, ~2.8 times). This suggests that protein-protein reproducibility may influence mRNA-protein correlation more than mRNA-mRNA reproducibility does but that 18 cell lines is not sufficient to obtain a robust estimate of protein-protein reproducibility.

The Spearman correlation between aggregated protein reproducibility and CCLE transcriptomic reproducibility is 0.37 across 4,795 proteins. This suggests that there is some agreement between the reproducibility of proteins and transcripts and that, to some extent, proteins that are reproducibly measured are encoded by transcripts that are more reproducibly measured. To assess if both mRNA and protein reproducibility independently contribute to the variability of mRNA-protein correlation across all 13 studies, we used a linear model with the two factors as independent variables and mRNA-protein correlation as the dependent variable. We found that in all cases, the two factors together explained a higher proportion of variance than either factor alone ($p < 0.001$, likelihood ratio test). In the case of the CCLE study (used to calculate the mRNA reproducibility and one of the three studies used to calculate protein reproducibility), the two factors together explained 48% of the variance. For the 12 other studies, the two factors together explained ~14%–26% of the variance (Figure 6B). These observations suggest that the reproducibility in transcriptomic and proteomic data contribute strongly and somewhat independently to the variability observed in mRNA-protein correlation.

Figure 4. Aggregated protein reproducibility ranks partially explains the variable mRNA-protein correlation in 10 additional studies
(A–J) For studies without experimental proteomic replicates, boxplots showing the distributions of mRNA-protein correlation for proteins in each decile of the aggregated protein reproducibility ranks. (A)–(H) are the CPTAC tumor studies; (I) is the NCI-60 cancer cell lines study wherein protein quantification, used for computing the mRNA-protein correlation, is obtained from data-independent acquisition-based untargeted proteomics (SWATH-MS); and (J) is the healthy tissues study from the GTEx Consortium. Box plot details as in Figure 2.

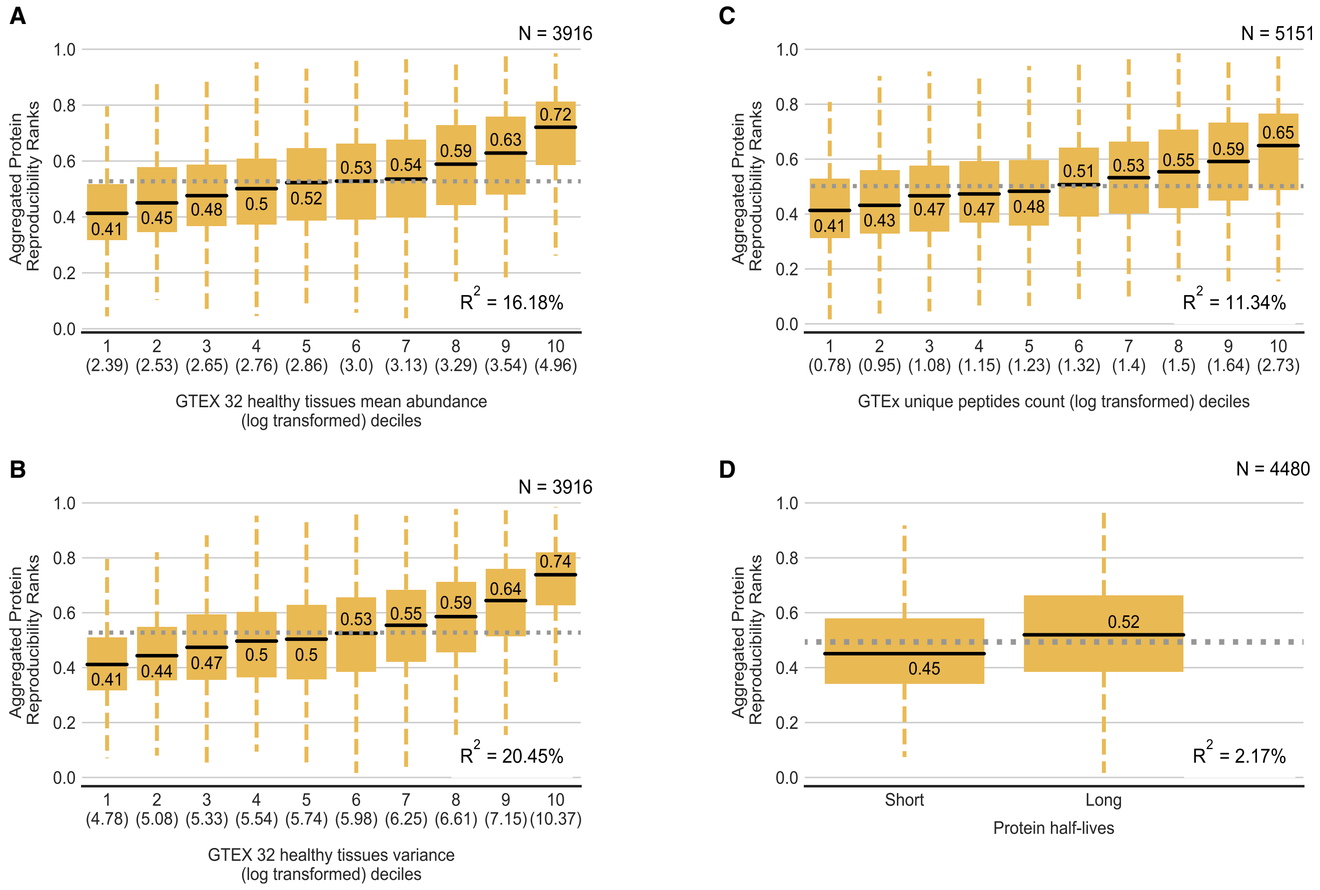

Figure 5. Protein reproducibility is mainly influenced by abundance, variance, and unique peptides and not protein half-lives

(A–C) Boxplots showing the distribution of aggregated protein reproducibility ranks for proteins binned according to protein abundance (A), variance (B), and number of unique peptides (C). Box plot details as in Figure 2.

(D) Boxplot showing the distribution of aggregated protein reproducibility ranks for proteins with short and long protein half-lives.

Metabolic pathways with higher-than-average mRNA-protein correlations may reflect differential reproducibility rather than differential post-transcriptional regulation

Previous work has found that certain pathways and processes are enriched in proteins that have higher- or lower-than-average mRNA-protein correlations. For instance, ribosomal subunits have been found to have consistently lower-than-average mRNA-protein correlations across multiple studies (Clark et al., 2019; Mertins et al., 2016; Zhang et al., 2014, 2016), while members of pathways related to amino acid metabolism have been found to have higher-than-average mRNA-protein correlation (Clark et al., 2019; Huang et al., 2021; Jarnuczak et al., 2021; Mertins et al., 2016; Zhang et al., 2014, 2016). This variation across functional groups has been attributed to differential post-transcriptional regulation. However, our observation that both protein-protein measurement reproducibility and mRNA-mRNA measurement reproducibility contribute significantly to the variation in mRNA-protein correlation across genes suggests an alternative explanation—some pathways may have higher- or lower-than-average mRNA-protein correlations simply because their component proteins are more reproducibly measured. To test this hypothesis, we first performed pathway enrichment analysis on the mRNA-protein correlations from the CCLE and ovarian datasets (STAR Methods; Figures 7 and S7). Consistent with previous studies, we observed that proteins with high mRNA-protein correlations are enriched in gene sets involved in environmental information processing and metabolic pathways, while proteins with low mRNA-protein correlations are enriched in annotations related to housekeeping protein complexes (Figure 7; Tables S3 and S4). To assess whether these enrichments could simply be attributed to variable reproducibility, we next performed pathway enrichment analysis on the CCLE and ovarian mRNA-protein correlation data after accounting for variation in protein-protein and mRNA-mRNA reproducibility (STAR Methods). We found in both studies that the "housekeeping" protein complexes were still identified as being enriched among proteins with lower-than-average mRNA-protein correlations but that the metabolic pathways were no longer enriched in proteins with higher-than-average mRNA-protein correlations (Figures 7 and S7; Tables S3 and S4). Other pathways with higher-than-average mRNA-protein correlations related to environmental information processing were also no longer significant after adjusting for reproducibility. This suggests that while large

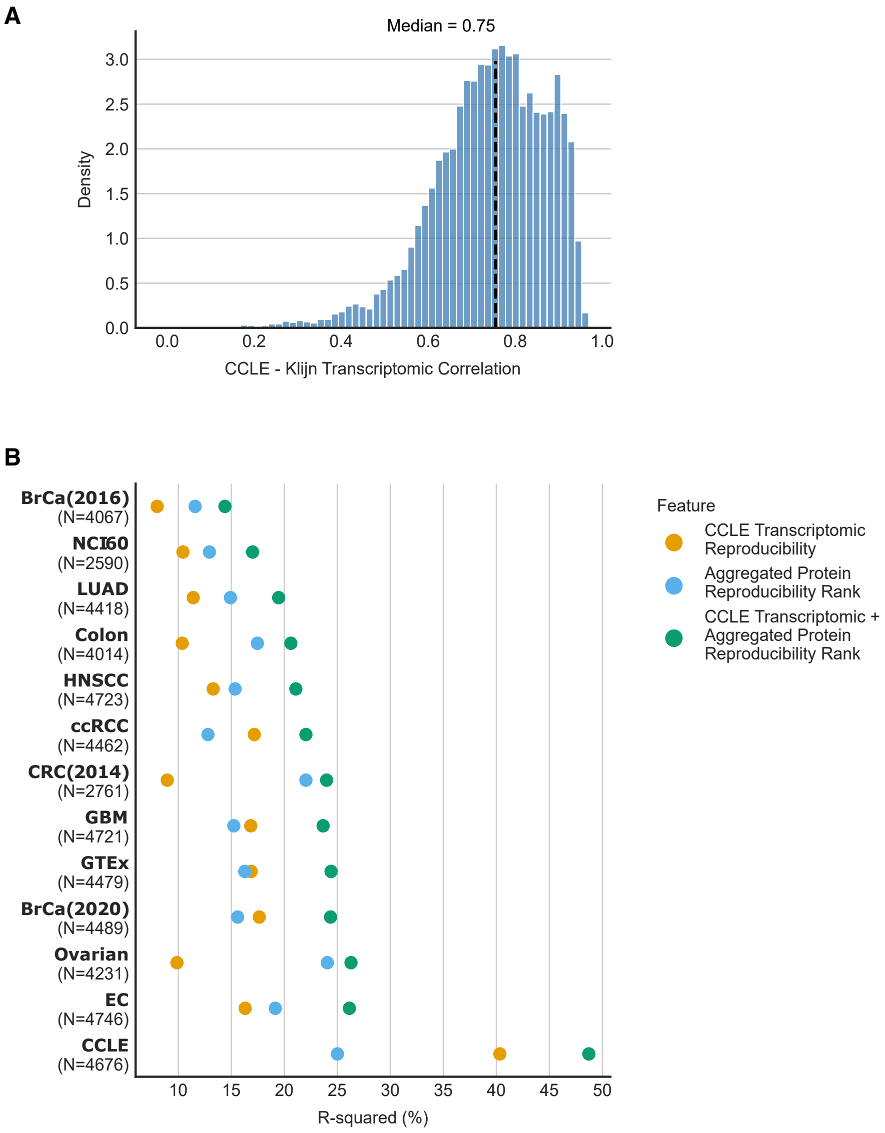

Figure 6. Transcriptomic reproducibility contributes to the variance in mRNA-protein correlation

(A) Histogram showing the distribution of the gene-wise correlation between experimental transcriptomic replicates of 382 cancer cell lines. The black line represents the median.

(B) For each of the 13 studies analyzed here, the R-squared obtained by regressing mRNA-protein correlation on transcriptomic reproducibility and aggregated protein reproducibility scores individually and in combination over the same set of proteins is shown in the dot plot. The number of proteins analyzed for each study is indicated in brackets below the study on the y axis.

housekeeping protein complexes such as the ribosome have lower-than-average mRNA-protein correlation that may be attributed to post-transcriptional mechanisms, the higher-than-average mRNA-protein correlation previously observed for metabolic pathways may simply reflect more reproducible measurements of their constituent proteins and transcripts.

DISCUSSION

Here, we have demonstrated that the reproducibility of protein and transcript measurements is a very significant factor in the observed correlations between mRNA and protein abundances. After taking this into account, we found that some pathways previously identified as having a high mRNA-protein correlation are likely just more reproducibly measured. We therefore suggest that conclusions about functional groups with higher or lower mRNA-protein correlations, especially with regard to the potential role played by post-transcriptional regulation, should be made only after accounting for variation in the measurement reproducibility of their constituent proteins. To this end, we have generated an aggregate protein reproducibility rank for each protein that can explain a significant amount of the variance across multiple proteogenomic studies and that may be useful for identifying those proteins that can be reliably and

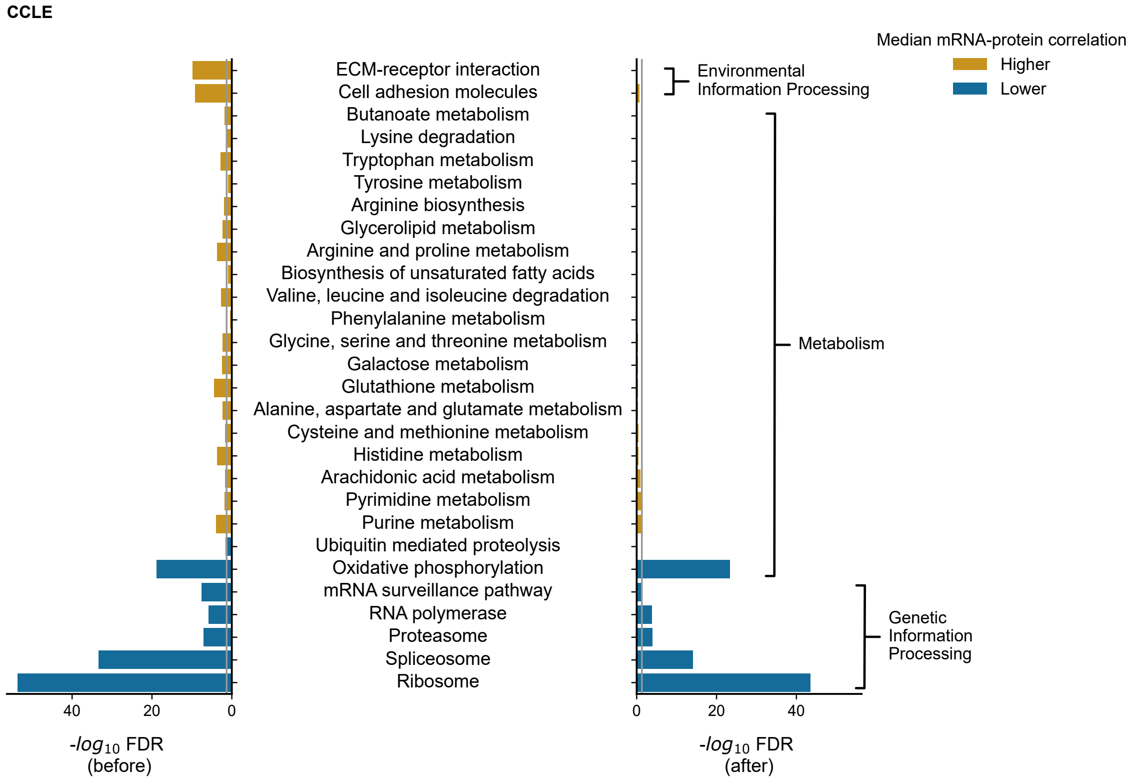

Figure 7. Metabolic pathways with higher-than-average mRNA-protein correlations may reflect differential reproducibility
Bar charts displaying the KEGG pathway enrichment analysis of the CCLE mRNA-protein correlation before (left) and after (right) accounting for protein-protein and mRNA-mRNA reproducibility. The $-\log_{10}$ of Benjamini-Hochberg false discovery rate (FDR)-corrected p values calculated using Mann-Whitney U test is used to assess enrichment for the pathway. For each bar chart, the gray line indicates the threshold considered for significant enrichment (FDR < 0.05). If the enrichment is below the threshold, then it is not considered significant. The bars are colored orange if the median mRNA-protein correlation of genes within the pathway is greater than the median mRNA-protein correlation of genes not in the pathway; otherwise, the bars are colored blue.

reproducibly measured by mass spectrometry. Such proteins may be more useful to assay in, e.g., diagnostic panels.

Recently, there have been a number of attempts to predict protein abundances from transcriptomic data that have achieved modest success (Barzine et al., 2020; Fortelny et al., 2017; Li et al., 2019; Yang et al., 2020). We found here that proteins that are more reproducibly measured across experimental replicates are better predicted using machine-learning. This suggests that one of the factors limiting the accuracy of machine-learning methods to predict protein abundances is that the protein abundance measurements themselves are not reproducible. It may therefore be worth evaluating future methods on the subset of proteins that can be reproducibly measured.

Limitations of the study

Our emphasis here has been on understanding how variability in the measurements of individual proteins can influence the mRNA-protein correlations observed in published tumor proteogenomic studies. We have shown that proteins/transcripts that are more reproducibly measured tend to have higher mRNA-protein correlations, and we have identified a number of factors (e.g., protein abundance) that influence variation in measurement reproducibility. There are of course additional factors that influence the global reproducibility of proteomes and transcriptomes quantified from "replicates" of the same sample. These include real biological variation (e.g., tumor heterogeneity resulting in two samples of the same tumor having different profiles) and technical variation (e.g., variation in sample preparation between different runs of the same sample). We have not been able to address how much of the variance in the measurements of individual proteins can be attributed to these global factors. It is likely that reducing these sources of global variation, e.g., through automated sample preparation, will improve the overall reproducibility of protein measurements. We note also that our analyses do not reflect the best possible reproducibility of proteomic and transcriptomic measurements, but rather they reflect the reproducibility observed in existing large-scale proteogenomic datasets. Indeed, we see that more recent proteogenomic studies have higher mRNA-protein correlations, suggesting that methodological improvements are already reducing the sources of noise in these approaches.

Our results from analyzing the CCLE dataset, where the replicate correlation is highest, give what is likely the most realistic assessment of proteomic reproducibility using modern MS pipelines. The replicates in the CCLE study were generated by the same lab, using the same methodology, 1 year apart. In contrast, the ovarian cancer study contains replicates generated in different labs (introducing significant measurement

heterogeneity), while the colon cancer study makes use of replicates generated using two entirely different MS approaches (label free versus TMT). Consequently, these likely represent lower-bound estimates of the reproducibility that can be observed using modern MS proteomic pipelines. Nonetheless, they likely reasonably approximate the non-biological heterogeneity observed between transcriptomes and proteomes in the studies analyzed, where mRNA and proteins are quantified separately using orthogonal techniques.

In the case of the CCLE data, we have a small number (18) of samples with replicate proteomic profiles available and a larger number (382) with replicate transcriptomes. However, only 8 samples have both replicate transcriptomes and replicate proteomes. Having a larger number of samples with both replicate proteomes and replicate transcriptomes would allow us to better estimate the actual correlation between mRNA and protein across samples after correction for measurement error, as has previously been done to estimate the true "within sample" mRNA-protein correlation in yeast (Csárdi et al., 2015; Spearman, 1904).

Here, we have shown that a number of factors measured using the GTEx dataset, including measured protein abundance, measured protein variance, and measured unique peptides, are associated with proteomic reproducibility in cancer studies. Across studies, a model that incorporates all three factors outperformed models using each variable alone. However, this may be because all three represent imperfect measurements of the same underlying variable—real average protein abundance. Previous work has demonstrated that statistical modeling that integrates multiple mRNA and protein datasets and explicitly takes into account different sources of noise and error can be used to provide improved estimates of mRNA-protein correlation within samples (Csárdi et al., 2015). As additional studies with proteomic replicates and transcriptomic replicates become available, it may be possible to develop improved models that provide more reliable estimates of protein reproducibility and the factors that influence it. Such estimates could be improved through the incorporation of additional estimates of average protein abundance and variation (e.g., from Wang et al., 2019).

STAR★METHODS

Detailed methods are provided in the online version of this paper and include the following:

- KEY RESOURCES TABLE
- RESOURCE AVAILABILITY
  - Lead contact
  - Materials availability
  - Data and code availability
- METHOD DETAILS
  - Data collection
  - Pre-processing proteomic and transcriptomic profiles
  - Computation of correlation coefficient
  - Assessing proteomic and transcriptomic reproducibility
  - Computation of deciles
  - Protein complex membership
  - Protein half-lives
  - Rank aggregation
  - Linear regression models
  - Rank regression
  - Pathway enrichment analysis
- QUANTIFICATION AND STATISTICAL ANALYSIS

SUPPLEMENTAL INFORMATION

Supplemental information can be found online at https://doi.org/10.1016/j.crmeth.2022.100288.

ACKNOWLEDGMENTS

S.R.U. was funded through the School of Computer Science, University College Dublin, and C.J.R. was funded by Irish Research Council Laureate Awards 2017/2018. We thank Dr. Dirk Fey, Dr. Giorgio Oliviero, Dr. Luis Iglesias Martinez, and members of the Ryan lab for careful reading of the manuscript and helpful feedback. We also thank Dr. Theodoros Roumeliotis for suggesting protein variance as a factor influencing protein reproducibility.

AUTHOR CONTRIBUTIONS

Conceptualization, S.R.U. and C.J.R.; methodology, S.R.U. and C.J.R.; formal analysis, S.R.U.; data curation, S.R.U.; writing – original draft, S.R.U. and C.J.R.; writing – review and editing, C.J.R.; visualization, S.R.U.; supervision, C.J.R.; funding acquisition, C.J.R.

DECLARATION OF INTERESTS

The authors declare no competing interests.

Received: February 12, 2022
Revised: July 14, 2022
Accepted: August 16, 2022
Published: September 8, 2022

REFERENCES

't Hoen, P.A.C., Friedländer, M.R., Almlöf, J., Sammeth, M., Pulyakhina, I., Anvar, S.Y., Laros, J.F.J., Buermans, H.P.J., Karlberg, O., Brännvall, M., et al. (2013). Reproducibility of high-throughput mRNA and small RNA sequencing across laboratories. Nat. Biotechnol. *31*, 1015–1022.

Aslam, J.A., and Montague, M. (2001). Models for metasearch. In Proceedings of the 24th Annual International ACM SIGIR Conference on Research and Development in Information Retrieval (Association for Computing Machinery), pp. 276–284.

Barzine, M.P., Freivalds, K., Wright, J.C., Opmanis, M., Rituma, D., Ghavidel, F.Z., Jarnuczak, A.F., Celms, E., Čerāns, K., Jonassen, I., et al. (2020). Using deep learning to extrapolate protein expression measurements. Proteomics *20*, e2000009.

Buccitelli, C., and Selbach, M. (2020). mRNAs, proteins and the emerging principles of gene expression control. Nat. Rev. Genet. *21*, 630–644.

Cancer Genome Atlas Network (2012). Comprehensive molecular characterization of human colon and rectal cancer. Nature *487*, 330–337.

Cancer Genome Atlas Research Network (2011). Integrated genomic analyses of ovarian carcinoma. Nature *474*, 609–615.

Casey, T.M., Khan, J.M., Bringans, S.D., Koudelka, T., Takle, P.S., Downs, R.A., Livk, A., Syme, R.A., Tan, K.-C., and Lipscombe, R.J. (2017). Analysis of reproducibility of proteome coverage and quantitation using isobaric mass tags (iTRAQ and TMT). J. Proteome Res. *16*, 384–392.

Cerami, E., Gao, J., Dogrusoz, U., Gross, B.E., Sumer, S.O., Aksoy, B.A., Jacobsen, A., Byrne, C.J., Heuer, M.L., Larsson, E., et al. (2012). The cBio cancer

genomics portal: an open platform for exploring multidimensional cancer genomics data. Cancer Discov. *2*, 401–404.

Ciriello, G., Gatza, M.L., Beck, A.H., Wilkerson, M.D., Rhie, S.K., Pastore, A., Zhang, H., McLellan, M., Yau, C., Kandoth, C., et al. (2015). Comprehensive molecular portraits of invasive lobular breast cancer. Cell *163*, 506–519.

Clark, D.J., Dhanasekaran, S.M., Petralia, F., Pan, J., Song, X., Hu, Y., da Veiga Leprevost, F., Reva, B., Lih, T.-S.M., Chang, H.-Y., et al. (2019). Integrated proteogenomic characterization of clear cell renal cell carcinoma. Cell *179*, 964–983.e31.

Csárdi, G., Franks, A., Choi, D.S., Airoldi, E.M., and Drummond, D.A. (2015). Accounting for experimental noise reveals that mRNA levels, amplified by post-transcriptional processes, largely determine steady-state protein levels in yeast. PLoS Genet. *11*, e1005206.

Dou, Y., Kawaler, E.A., Cui Zhou, D., Gritsenko, M.A., Huang, C., Blumenberg, L., Karpova, A., Petyuk, V.A., Savage, S.R., Satpathy, S., et al. (2020). Proteogenomic characterization of endometrial carcinoma. Cell *180*, 729–748.e26.

Dwork, C., Kumar, R., Naor, M., and Sivakumar, D. (2001). Rank aggregation methods for the Web. In Proceedings of the 10th International Conference on World Wide Web (Association for Computing Machinery)), pp. 613–622.

Ellis, M.J., Gillette, M., Carr, S.A., Paulovich, A.G., Smith, R.D., Rodland, K.K., Townsend, R.R., Kinsinger, C., Mesri, M., Rodriguez, H., et al. (2013). Connecting genomic alterations to cancer biology with proteomics: the NCI clinical proteomic tumor analysis Consortium. Cancer Discov. *3*, 1108–1112.

Fortelny, N., Overall, C.M., Pavlidis, P., and Freue, G.V.C. (2017). Can we predict protein from mRNA levels? Nature *547*, E19–E20.

Franks, A., Airoldi, E., and Slavov, N. (2017). Post-transcriptional regulation across human tissues. PLoS Comput. Biol. *13*, e1005535.

Gao, J., Aksoy, B.A., Dogrusoz, U., Dresdner, G., Gross, B., Sumer, S.O., Sun, Y., Jacobsen, A., Sinha, R., Larsson, E., et al. (2013). Integrative analysis of complex cancer genomics and clinical profiles using the cBioPortal. Sci. Signal. *6*, l1.

Ghandi, M., Huang, F.W., Jané-Valbuena, J., Kryukov, G.V., Lo, C.C., McDonald, E.R., 3rd, Barretina, J., Gelfand, E.T., Bielski, C.M., Li, H., et al. (2019). Next-generation characterization of the cancer cell line encyclopedia. Nature *569*, 503–508.

Gillette, M.A., Satpathy, S., Cao, S., Dhanasekaran, S.M., Vasaikar, S.V., Krug, K., Petralia, F., Li, Y., Liang, W.-W., Reva, B., et al. (2020). Proteogenomic characterization reveals therapeutic vulnerabilities in lung adenocarcinoma. Cell *182*, 200–225.e35.

Giurgiu, M., Reinhard, J., Brauner, B., Dunger-Kaltenbach, I., Fobo, G., Frishman, G., Montrone, C., and Ruepp, A. (2019). CORUM: the comprehensive resource of mammalian protein complexes-2019. Nucleic Acids Res. *47*, D559–D563.

Gonçalves, E., Fragoulis, A., Garcia-Alonso, L., Cramer, T., Saez-Rodriguez, J., and Beltrao, P. (2017). Widespread post-transcriptional attenuation of genomic copy-number variation in cancer. Cell Syst. *5*, 386–398.e4.

Guo, T., Luna, A., Rajapakse, V.N., Koh, C.C., Wu, Z., Liu, W., Sun, Y., Gao, H., Menden, M.P., Xu, C., et al. (2019). Quantitative proteome landscape of the NCI-60 cancer cell lines. iScience *21*, 664–680.

Harris, C.R., Millman, K.J., van der Walt, S.J., Gommers, R., Virtanen, P., Cournapeau, D., Wieser, E., Taylor, J., Berg, S., Smith, N.J., et al. (2020). Array programming with NumPy. Nature *585*, 357–362.

Huang, C., Chen, L., Savage, S.R., Eguez, R.V., Dou, Y., Li, Y., da Veiga Leprevost, F., Jaehnig, E.J., Lei, J.T., Wen, B., et al. (2021). Proteogenomic insights into the biology and treatment of HPV-negative head and neck squamous cell carcinoma. Cancer Cell *39*, 361–379.e16.

Hunter, J.D. (2007). Matplotlib: a 2D Graphics environment. Comput. Sci. Eng. *9*, 90–95.

Jarnuczak, A.F., Najgebauer, H., Barzine, M., Kundu, D.J., Ghavidel, F., Perez-Riverol, Y., Papatheodorou, I., Brazma, A., and Vizcaíno, J.A. (2021). An integrated landscape of protein expression in human cancer. Sci. Data *8*, 115.

Jiang, L., Wang, M., Lin, S., Jian, R., Li, X., Chan, J., Dong, G., Fang, H., Robinson, A.E., GTEx Consortium, et al. (2020). A quantitative proteome map of the human body. Cell *183*, 269–283.e19.

Kanehisa, M. (2019). Toward understanding the origin and evolution of cellular organisms. Protein Sci. *28*, 1947–1951.

Kanehisa, M., and Goto, S. (2000). KEGG: kyoto encyclopedia of genes and genomes. Nucleic Acids Res. *28*, 27–30.

Kanehisa, M., Furumichi, M., Sato, Y., Ishiguro-Watanabe, M., and Tanabe, M. (2021). KEGG: integrating viruses and cellular organisms. Nucleic Acids Res. *49*, D545–D551.

Klijn, C., Durinck, S., Stawiski, E.W., Haverty, P.M., Jiang, Z., Liu, H., Degenhardt, J., Mayba, O., Gnad, F., Liu, J., et al. (2015). A comprehensive transcriptional portrait of human cancer cell lines. Nat. Biotechnol. *33*, 306–312.

Kolde, R., Laur, S., Adler, P., and Vilo, J. (2012). Robust rank aggregation for gene list integration and meta-analysis. Bioinformatics *28*, 573–580.

Krug, K., Jaehnig, E.J., Satpathy, S., Blumenberg, L., Karpova, A., Anurag, M., Miles, G., Mertins, P., Geffen, Y., Tang, L.C., et al. (2020). Proteogenomic landscape of breast cancer tumorigenesis and targeted therapy. Cell *183*, 1436–1456.e31.

Li, H., Siddiqui, O., Zhang, H., and Guan, Y. (2019). Joint learning improves protein abundance prediction in cancers. BMC Biol. *17*, 107.

Li, J.J., Bickel, P.J., and Biggin, M.D. (2014). System wide analyses have underestimated protein abundances and the importance of transcription in mammals. PeerJ *2*, e270.

Lindgren, C.M., Adams, D.W., Kimball, B., Boekweg, H., Tayler, S., Pugh, S.L., and Payne, S.H. (2021). Simplified and unified access to cancer proteogenomic data. J. Proteome Res. *20*, 1902–1910.

Liu, Y., Beyer, A., and Aebersold, R. (2016). On the dependency of cellular protein levels on mRNA abundance. Cell *165*, 535–550.

Marioni, J.C., Mason, C.E., Mane, S.M., Stephens, M., and Gilad, Y. (2008). RNA-seq: an assessment of technical reproducibility and comparison with gene expression arrays. Genome Res. *18*, 1509–1517.

McKinney, W. (2011). pandas: a foundational Python library for data analysis and statistics. Python for High Performance and Scientific Computing *14*.

Mertins, P., Mani, D.R., Ruggles, K.V., Gillette, M.A., Clauser, K.R., Wang, P., Wang, X., Qiao, J.W., Cao, S., Petralia, F., et al. (2016). Proteogenomics connects somatic mutations to signalling in breast cancer. Nature *534*, 55–62.

Nusinow, D.P., and Gygi, S.P. (2020). A Guide to the Quantitative Proteomic Profiles of the Cancer Cell Line Encyclopedia (BioRxiv). https://doi.org/10.1101/2020.02.03.932384.

Nusinow, D.P., Szpyt, J., Ghandi, M., Rose, C.M., McDonald, E.R., 3rd, Kalocsay, M., Jané-Valbuena, J., Gelfand, E., Schweppe, D.K., Jedrychowski, M., et al. (2020). Quantitative proteomics of the cancer cell line encyclopedia. Cell *180*, 387–402.e16.

Ősz, Á., Lánczky, A., and Győrffy, B. (2021). Survival analysis in breast cancer using proteomic data from four independent datasets. Sci. Rep. *11*, 16787.

Payne, S.H. (2015). The utility of protein and mRNA correlation. Trends Biochem. Sci. *40*, 1–3.

Ryan, C.J., Kennedy, S., Bajrami, I., Matallanas, D., and Lord, C.J. (2017). A compendium of Co-regulated protein complexes in breast cancer reveals collateral loss events. Cell Syst. *5*, 399–409.e5.

Seabold, S., and Perktold, J. (2010). Statsmodels: econometric and statistical modeling with python. In Proceedings of the 9th Python in Science Conference, p. 61.

SEQC/MAQC-III Consortium (2014). A comprehensive assessment of RNA-seq accuracy, reproducibility and information content by the Sequencing Quality Control Consortium. Nat. Biotechnol. *32*, 903–914.

Shenoy, A., Belugali Nataraj, N., Perry, G., Loayza Puch, F., Nagel, R., Marin, I., Balint, N., Bossel, N., Pavlovsky, A., Barshack, I., et al. (2020). Proteomic patterns associated with response to breast cancer neoadjuvant treatment. Mol. Syst. Biol. *16*, e9443.

Spearman, C. (1904). The proof and measurement of association between two things. Am. J. Psychol. *15*, 72–101.

Stuart, J.M., Segal, E., Koller, D., and Kim, S.K. (2003). A gene-coexpression network for global discovery of conserved genetic modules. Science *302*, 249–255.

Tabb, D.L., Vega-Montoto, L., Rudnick, P.A., Variyath, A.M., Ham, A.-J.L., Bunk, D.M., Kilpatrick, L.E., Billheimer, D.D., Blackman, R.K., Cardasis, H.L., et al. (2010). Repeatability and reproducibility in proteomic identifications by liquid chromatography-tandem mass spectrometry. J. Proteome Res. *9*, 761–776.

Taggart, J.C., Zauber, H., Selbach, M., Li, G.-W., and McShane, E. (2020). Keeping the proportions of protein complex components in check. Cell Syst. *10*, 125–132.

Vasaikar, S., Huang, C., Wang, X., Petyuk, V.A., Savage, S.R., Wen, B., Dou, Y., Zhang, Y., Shi, Z., Arshad, O.A., et al. (2019). Proteogenomic analysis of human colon cancer reveals new therapeutic opportunities. Cell *177*, 1035–1049.e19.

Vasaikar, S.V., Straub, P., Wang, J., and Zhang, B. (2018). LinkedOmics: analyzing multi-omics data within and across 32 cancer types. Nucleic Acids Res. *46*, D956–D963.

Virtanen, P., Gommers, R., Oliphant, T.E., Haberland, M., Reddy, T., Cournapeau, D., Burovski, E., Peterson, P., Weckesser, W., Bright, J., et al. (2020). SciPy 1.0: fundamental algorithms for scientific computing in Python. Nat. Methods *17*, 261–272.

Vogel, C., and Marcotte, E.M. (2012). Insights into the regulation of protein abundance from proteomic and transcriptomic analyses. Nat. Rev. Genet. *13*, 227–232.

Wang, D., Eraslan, B., Wieland, T., Hallström, B., Hopf, T., Zolg, D.P., Zecha, J., Asplund, A., Li, L.-H., Meng, C., et al. (2019). A deep proteome and transcriptome abundance atlas of 29 healthy human tissues. Mol. Syst. Biol. *15*, e8503.

Wang, J., Ma, Z., Carr, S.A., Mertins, P., Zhang, H., Zhang, Z., Chan, D.W., Ellis, M.J.C., Townsend, R.R., Smith, R.D., et al. (2017). Proteome profiling outperforms transcriptome profiling for coexpression based gene function prediction. Mol. Cell. Proteomics *16*, 121–134.

Wang, L.-B., Karpova, A., Gritsenko, M.A., Kyle, J.E., Cao, S., Li, Y., Rykunov, D., Colaprico, A., Rothstein, J.H., Hong, R., et al. (2021). Proteogenomic and metabolomic characterization of human glioblastoma. Cancer Cell *39*, 509–528.e20.

Waskom, M.L. (2021). seaborn: statistical data visualization. Journal of Open Source Software *6*, 3021.

Wu, L., Candille, S.I., Choi, Y., Xie, D., Jiang, L., Li-Pook-Than, J., Tang, H., and Snyder, M. (2013). Variation and genetic control of protein abundance in humans. Nature *499*, 79–82.

Yang, M., Petralia, F., Li, Z., Li, H., Ma, W., Song, X., Kim, S., Lee, H., Yu, H., Lee, B., et al. (2020). Community assessment of the predictability of cancer protein and phosphoprotein levels from genomics and transcriptomics. Cell Syst. *11*, 186–195.e9.

Zecha, J., Meng, C., Zolg, D.P., Samaras, P., Wilhelm, M., and Kuster, B. (2018). Peptide level turnover measurements enable the study of proteoform dynamics. Mol. Cell. Proteomics *17*, 974–992.

Zhang, B., Wang, J., Wang, X., Zhu, J., Liu, Q., Shi, Z., Chambers, M.C., Zimmerman, L.J., Shaddox, K.F., Kim, S., et al. (2014). Proteogenomic characterization of human colon and rectal cancer. Nature *513*, 382–387.

Zhang, H., Liu, T., Zhang, Z., Payne, S.H., Zhang, B., McDermott, J.E., Zhou, J.-Y., Petyuk, V.A., Chen, L., Ray, D., et al. (2016). Integrated proteogenomic characterization of human high-Grade serous ovarian cancer. Cell *166*, 755–765.

STAR★METHODS

KEY RESOURCES TABLE

REAGENT or RESOURCE	SOURCE	IDENTIFIER
Deposited data		
cBioPortal	Cerami et al. (2012); Gao et al. (2013)	https://www.cbioportal.org/; RRID:SCR_014555
Cancer Dependency Map (DepMap) 20Q4	Ghandi et al. (2019)	https://depmap.org/portal/ccle/; https://figshare.com/articles/dataset/DepMap_20Q4_Public/13237076; RRID:SCR_017655
LinkedOmics	Vasaikar et al. (2018)	http://www.linkedomics.org/
CPTAC Python API	Lindgren et al. (2021)	https://pypi.org/project/cptac/
CORUM 3.0	Giurgiu et al. (2019)	http://mips.helmholtz-muenchen.de/corum/; RRID:SCR_002254
KEGG Pathway	Kanehisa (2019); Kanehisa and Goto (2000); Kanehisa et al. (2021)	https://www.genome.jp/kegg/pathway.html; RRID:SCR_018145
Colorectal cancer transcriptomics	Cancer Genome Atlas Network (2012)	https://cbioportal-datahub.s3.amazonaws.com/coadread_tcga_pub.tar.gz
Colorectal cancer proteomics	Zhang et al. (2014)	Published supplemental Table S4
Ovarian cancer transcriptomics	Cancer Genome Atlas Research Network (2011)	http://gdac.broadinstitute.org/runs/stddata__2016_01_28/data/OV/20160128/gdac.broadinstitute.org_OV.mRNA_Preprocess_Median.Level_3.2016012800.0.0.tar.gz
Ovarian cancer proteomics	Zhang et al. (2016)	Published supplemental Table S2
Breast Cancer (2016) transcriptomics	Ciriello et al. (2015)	https://cbioportal-datahub.s3.amazonaws.com/brca_tcga_pub2015.tar.gz
Breast Cancer (2016) proteomics	Mertins et al. (2016)	Published supplemental Table S3
Colon Cancer	Vasaikar et al. (2019)	http://linkedomics.org/cptac-colon/
Clear cell renal carcinoma	Clark et al. (2019)	https://pypi.org/project/cptac/
Breast Cancer (2020)	Krug et al. (2020)	https://pypi.org/project/cptac/
Endometrial Cancer	Dou et al. (2020)	https://pypi.org/project/cptac/
Lung Adenocarcinoma	Gillette et al. (2020)	https://pypi.org/project/cptac/
Head and Neck Squamous Cell Carcinoma	Huang et al. (2021)	https://pypi.org/project/cptac/
Glioblastoma	Wang et al. (2021)	https://pypi.org/project/cptac/
NCI60 cancer cell lines	Guo et al. (2019)	Published supplemental Tables S6 and S1
Cancer Cell Line Encyclopedia (CCLE) transcriptomics	Ghandi et al. (2019)	https://depmap.org/portal/ccle/; RRID:SCR_013836
CCLE proteomics	Nusinow et al. (2020)	Published supplemental Tables S2 and S3; https://gygi.hms.harvard.edu/publications/ccle.html
GTEx healthy tissues	Jiang et al. (2020)	Published supplemental Tables S3 and S4
RNA-seq of 675 commonly used human cancer cell lines	Klijn et al. (2015)	ArrayExpress: E-MTAB-2706
Protein half-life	Zecha et al. (2018)	Published supplemental Table S3
NCI CPTAC DREAM Proteogenomics challenge prediction scores of the best performing model (Team Guan)	Yang et al. (2020)	https://heidelberg.shinyapps.io/proteoexplorer/

(Continued on next page)

Continued

REAGENT or RESOURCE	SOURCE	IDENTIFIER
Software and algorithms		
All analysis code	This study	https://github.com/cancergenetics/limitations_of_omics_reproducibility; https://doi.org/10.5281/zenodo.6956546
Python version 3.8	Python Software Foundation	https://www.python.org/; RRID:SCR_008394
Pandas 1.2.5	McKinney (2011)	https://pandas.pydata.org/; RRID:SCR_018214
Numpy 1.20.2	Harris et al. (2020)	https://numpy.org/; RRID:SCR_008633
StatsModels 0.12.2	Seabold and Perktold (2010)	https://www.statsmodels.org/stable/index.html; RRID:SCR_016074
SciPy 1.7.1	Virtanen et al. (2020)	https://www.scipy.org/; RRID:SCR_008058
Matplotlib 3.3.4	Hunter (2007)	https://matplotlib.org/; RRID: SCR_008624
Seaborn 0.11.0	Waskom (2021)	https://seaborn.pydata.org/; RRID:SCR_018132

RESOURCE AVAILABILITY

Lead contact

Further information and requests for resources should be directed to and will be fulfilled by the lead contact, Colm Ryan (colm.ryan@ucd.ie).

Materials availability

This study did not generate new materials.

Data and code availability

- This paper analysed existing, publicly available data. The accession numbers for the datasets are listed in the key resources table.
- All original code has been deposited at Github and Zenodo and is publicly available as of the date of publication. DOIs are listed in the key resources table.
- Any additional information required to reanalyse the data reported in this work paper is available from the lead contact upon request.

METHOD DETAILS

Data collection

The datasets analysed were downloaded from the links provided in the key resources table.

For studies (Clark et al., 2019; Dou et al., 2020; Gillette et al., 2020; Huang et al., 2021; Krug et al., 2020; Wang et al., 2021) both the transcriptomic and proteomic profiles were obtained from the CPTAC API (Lindgren et al., 2021). For colorectal (Zhang et al., 2014) and breast cancer (Mertins et al., 2016) studies, the transcriptomic data were downloaded from cBioPortal while proteomic data was obtained from the supplemental materials. For the ovarian cancer study (Zhang et al., 2016), the transcriptomic data were downloaded from the https://gdac.broadinstitute.org/ and proteomic data from the supplemental materials. For colon cancer (Vasaikar et al., 2019), GTEX (Jiang et al., 2020) and NCI60 (Guo et al., 2019) cancer cell lines studies, both the transcriptomic and proteomic data were obtained from the supplemental tables. For CCLE study, the transcriptomic data was downloaded from the cancer dependency map portal (https://depmap.org/portal/ccle/) and proteomic data was downloaded from the supplemental materials.

Pre-processing proteomic and transcriptomic profiles

Proteomics and transcriptomics data were obtained from the studies listed in the Key resources table. The proteomics datasets contained a considerable number of missing values, identified as NaNs in most studies or 0s in (Zhang et al., 2014). Within each study we restricted our analyses to proteins that were measured in at least 80% of samples. The same filtering was applied to transcriptomics, requiring transcripts to be measured in 80% of samples. In some datasets, multiple protein isoforms from the same gene were available, we aggregated these using the mean to calculate a 'gene level' summary.

The CCLE study repeatedly profiled two 10-plexes (18 cell lines) one year apart in order to assess the reproducibility of the proteomic profiling. These replicates are used to perform the assessment of the reproducibility of protein measurements presented in Figure 1. In addition to these 18 cell lines, 3 cell lines were screened in duplicate as part of standard 10-plex runs. As suggested

in the CCLE guide (Nusinow and Gygi, 2020) for these three cell lines we selected the profiles which correlate best with the transcriptomic data for our analyses here.

Computation of correlation coefficient

All data was processed through the standard pipeline described above before computing correlation. Correlation between (i) mRNA-protein, (ii) protein-protein and (iii) mRNA-mRNA was computed using the Spearman rank correlation. For each protein in each study, samples with missing values were ignored when computing the correlation.

Assessing proteomic and transcriptomic reproducibility

The quantitative proteomics of the CCLE (Nusinow et al., 2020) data contained three replicates of the proteomic profiles. In the first year, 18 cell lines (two 10-plexes) were quantified (R1). The same cell lines were quantified twice (R2, R3) the following year. The correlation between replicates: R1-R2, R1-R3 and R2-R3 were 0.7, 0.71 and 0.88 respectively. We chose to use the R1 and R3 proteomic profiles to compute the replicate correlation as R1-R3 has the median correlation out of the three replicate pairs.

To assess the reproducibility of transcriptomic data we considered two studies that had quantified transcripts in tumour-derived cell lines. One of the studies chosen was the CCLE transcriptomic study for which we have previously assessed the mRNA-protein correlation. The CCLE transcriptomic study (Ghandi et al., 2019) had profiled 1076 and (Klijn et al., 2015) had profiled 675 cancer cell lines using RNA-Seq. These two studies had quantified the transcripts in different labs in different years. However, the two studies had 382 cell lines and 13,226 genes in common. The transcriptomic reproducibility was computed using the Spearman rank correlation coefficient for the transcriptomic measurements across the 382 common cell lines of the studies. The standard pipeline for pre-processing was applied before assessing the reproducibility of the transcriptomic studies.

While the CCLE transcriptomic reproducibility was computed using 382 cell lines, the CCLE proteomic reproducibility was computed using 18 cell lines only. The common cell lines between the transcriptomic and proteomic replicates were <10. Therefore, to compare the predictive power of transcriptomic reproducibility and proteomic reproducibility in explaining the variation in mRNA-protein correlation of the different studies, the transcriptomic reproducibility was computed for 18 random cell lines over 100 iterations. The transcriptomic reproducibility was then used to predict the mRNA-protein correlation of the thirteen proteogenomic studies. For each study, the mean R^2 obtained across all 100 random cell line selections was then used to compare the predictive power of transcriptomic reproducibility and proteomic reproducibility over the same number of proteins.

Computation of deciles

Deciles were computed using the pandas qcut method. Each decile contains ~10% of the overall number of items to be stratified. In some cases, due to ties, these deciles are not uniformly sized.

Protein complex membership

Information on protein complex membership was obtained from CORUM (Giurgiu et al., 2019) (all complexes data). A protein was marked as a protein complex subunit if it is identified in CORUM data.

Protein half-lives

The half-lives of proteins were obtained from (Zecha et al., 2018) study. The median half-life of all proteins from the list was computed. Proteins with half-lives > median were encoded to have ‘long’ half-life while the others were encoded to have ‘short’ half-life.

Rank aggregation

For each of the three proteomic studies with replicates (ovarian, colon, CCLE) ranks were assigned based on increasing correlation and normalized by dividing over the total number of proteins in the dataset. Only proteins that were measured in 2 out of the 3 datasets were considered for the aggregated list. For proteins measured in only 2 studies, we imputed the third normalised rank as 0.5. For all proteins, we then computed the mean rank as the aggregated rank of the protein (Figure S2A).

We compared the aggregated list of proteins obtained through our method of aggregation (Figure S2A) with other aggregated lists which we calculated using other algorithms - robust rank aggregation (Kolde et al., 2012), Stuart (Stuart et al., 2003), BordaFuse (Aslam and Montague, 2001) and, Markov Chain Aggregator (MC4) (Dwork et al., 2001). To assess the performance of different aggregation methods, we used linear models wherein the mRNA-protein correlation of the three studies containing replicate proteomic profiles was regressed on the different aggregated lists of protein reproducibility. The aggregated list using our ‘average normalized rank’ approach could best explain the variation in mRNA-protein correlation in the colorectal cancer and CCLE studies, while the BordaFuse method best explained the variation in the ovarian cancer study (Figure S2B), followed by our approach. As our ‘average normalized rank’ approach overall has the highest R-squared, we chose this method to aggregate the correlations of proteomic replicate profiles.

Linear regression models

All linear regression was carried out using the statsmodel package in Python.

Assessing the relationship between protein-protein reproducibility, mRNA-mRNA reproducibility, and mRNA-protein correlation (Figure 6B)

To understand the variance in mRNA-protein correlation explained by protein-protein reproducibility and mRNA-mRNA reproducibility we used three different linear models given by the equations -

- Protein-protein reproducibility only: $c(g) = \alpha + \beta * p_a(g)$
- mRNA-mRNA reproducibility only: $c(g) = \alpha + \beta * m(g)$
- Protein-protein reproducibility and mRNA-mRNA reproducibility: $c(g) = \alpha + \beta_1 * p(g) + \beta_2 * m(g)$

where $c(g)$ is the mRNA-protein correlation for each protein, $p_a(g)$ is the aggregated protein reproducibility rank for each protein, $m(g)$ is the mRNA-mRNA reproducibility of the corresponding transcript of each protein and the coefficients α, β, β_1 and β_2 are computed using the ordinary least squares regression method. For all the models, mRNA-protein correlation is assessed over the same set of proteins in each study. R^2 is used to assess the predictive power of the explanatory variables in explaining the variation of the response variable.

Assessing the relationship between protein-protein reproducibility and mRNA-protein correlation (Figure S1A)

To understand the variance in mRNA-protein correlation explained by protein complex membership and protein-protein reproducibility we used three different linear models given by the equations -

- Protein complex membership only: $c(g) = \alpha + \beta * pcm(g)$
- protein-protein reproducibility only: $c(g) = \alpha + \beta * p(g)$
- Protein complex membership and protein-protein reproducibility: $c(g) = \alpha + \beta_1 * pcm(g) + \beta_2 * p(g)$

where $c(g)$ is the mRNA-protein correlation for each protein, $pcm(g)$ is the protein complex membership for each protein, $p(g)$ is the protein-protein correlation for each protein and the coefficients α, β, β_1 and β_2 are computed using the ordinary least squares regression method. The protein complex membership is indicated as 1 if a protein is a protein complex member, else 0. For all the models, mRNA-protein correlation is assessed over the same set of proteins in each study. R^2 is used to assess the predictive power of the explanatory variables in explaining the variation of the response variable.

Assessing the ability of different aggregation approaches to rank protein-protein reproducibility (Figure S2B)

To identify the best aggregation method for protein-protein reproducibility, we compared the variance in mRNA-protein correlation explained by different aggregation methods using linear models given by the equations -

- Robust rank aggregation: $c(g) = \alpha + \beta * p_{rra}(g)$
- Stuart aggregation method: $c(g) = \alpha + \beta * p_{stuart}(g)$
- BordaFuse aggregation method: $c(g) = \alpha + \beta * p_{bf}(g)$
- Markov chain aggregator 4: $c(g) = \alpha + \beta * p_{mc4}(g)$
- Average normalized rank: $c(g) = \alpha + \beta * p_a(g)$

where $c(g)$ is the mRNA-protein correlation for each protein, $p_{rra}(g)$, $p_{stuart}(g)$, $p_{bf}(g)$, $p_{mc4}(g)$ and $p_a(g)$ are the aggregated protein reproducibility ranks computed using robust rank aggregation, Stuart, BordaFuse, Markov chain aggregator 4 and average normalized ranks respectively for each protein. The coefficients α and β are computed using the ordinary least squares regression method. For all the models, mRNA-protein correlation is assessed over the same set of proteins in each study. R^2 is used to assess the predictive power of the explanatory variables in explaining the variation of the response variable.

Comparing the ability if aggregated rank reproducibility to predict mRNA-protein correlation compared to reproducibility calculated in individual studies (Figure S3)

For each study, we compared four different models given by the equations -

- Ovarian protein reproducibility rank: $c(g) = \alpha + \beta * p_{ovarian}(g)$
- CCLE protein reproducibility rank: $c(g) = \alpha + \beta * p_{ccle}(g)$
- Colon protein reproducibility rank: $c(g) = \alpha + \beta * p_{colon}(g)$
- Aggregated protein reproducibility rank: $c(g) = \alpha + \beta * p_a(g)$

where $c(g)$ is the mRNA-protein correlation for each protein, $p_{ovarian}(g)$, $p_{ccle}(g)$, $p_{colon}(g)$ and $p_a(g)$ are the aggregated protein reproducibility computed using the ovarian, CCLE and colon proteomic replicates individually and collectively respectively for each protein. The coefficients α and β are computed using the ordinary least squares regression method. For all the models, mRNA-protein correlation is assessed over the same set of proteins in each study. R^2 is used to assess the predictive power of the explanatory variables in explaining the variation of the response variable.

Assessing the impact of protein measurement reproducibility on the accuracy of machine learning prediction of protein abundance (Figure S4C)

To understand the variation in protein prediction scores that can be explained by protein-protein reproducibility, we compared four different models on prediction scores of breast and ovarian tumour studies given by the equations -

- Ovarian protein reproducibility rank: $p_{scores}(g) = \alpha + \beta * p_{ovarian}(g)$
- CCLE protein reproducibility rank: $p_{scores}(g) = \alpha + \beta * p_{ccle}(g)$
- Colon protein reproducibility rank: $p_{scores}(g) = \alpha + \beta * p_{colon}(g)$
- Aggregated protein reproducibility rank: $p_{scores}(g) = \alpha + \beta * p_a(g)$

where $p_{scores}(g)$ is the prediction score that is the Pearson correlation between the predicted and actual protein abundance value obtained from the best predicting model in NCI CPTAC Proteogenomics DREAM challenge, $p_{ovarian}(g)$, $p_{ccle}(g)$, $p_{colon}(g)$ and $p_a(g)$ are the aggregated protein reproducibility computed using the ovarian, CCLE and colon proteomic replicates individually and collectively respectively for each protein. The coefficients α and β are computed using the ordinary least squares regression method. For all the models, protein reproducibility rank is assessed over the same set of proteins in each study. R^2 is used to assess the predictive power of the explanatory variables in explaining the variation of the response variable.

Assessing the impact of mRNA abundance, mRNA variance on the reproducibility of transcripts (Figure S5E)

To understand the variation in mRNA reproducibility explained by the potential factors (mRNA abundance, mRNA variance), we used two different linear models given by the equations -

- mRNA abundance only: $t(g) = \alpha + \beta * m_{abundance}(g)$
- mRNA variance only: $t(g) = \alpha + \beta * m_{variance}(g)$

where $t(g)$ is the transcript reproducibility correlation for each transcript, $m_{abundance}(g)$ is the mRNA mean abundance for each transcript obtained from CCLE transcriptomic data, $m_{variance}(g)$ is the variance of the mRNA abundance for each transcript obtained from CCLE transcriptomic data and the coefficients α and β are computed using the ordinary least squares regression method.

Assessing the impact of protein abundance, protein variance, unique peptides, protein half-lives and aggregated protein reproducibility on mRNA-protein correlation (Figure S6)

To understand the variance in mRNA-protein correlation explained by the factors (protein abundance, protein variance, unique peptides, and protein half-lives) influencing protein reproducibility, we used two different linear models given by the equations -

- Other factors ():

$$c(g) = \alpha + \beta_1 * p_{abundance}(g) + \beta_2 * p_{variance}(g) + \beta_3 * p_{peptides}(g) + \beta_4 * p_{half-lives-long}(g) + \beta_5 * p_{half-lives-short}(g)$$

- Aggregated protein reproducibility: $c(g) = \alpha + \beta * p_a(g)$

where $c(g)$ is the mRNA-protein correlation for each protein, $p_{abundance}(g)$ is the protein abundance for each protein obtained from the GTEx project, $p_{variance}(g)$ is the variance of the protein abundance for each protein obtained from the GTEx project, $p_{peptides}(g)$ is the number of unique peptides for each protein obtained from the GTEx project, $p_{half-lives-long}(g)$ and $p_{half-lives-short}(g)$ are the half-lives of each protein (long and short), $p_a(g)$ are the aggregated protein reproducibility computed using the ovarian, CCLE and colon proteomic replicates individually and collectively respectively for each protein and the coefficients: α, β, β_1, β_2, β_3, β_4 and β_5 are computed using the ordinary least squares regression method. For all the models, mRNA-protein correlation is assessed over the same set of proteins in each study. R^2 is used to assess the predictive power of the explanatory variables in explaining the variation of the response variable.

Rank regression

We used rank regression to assess the contribution of various factors (protein abundance, unique peptides, and protein half-lives) to explaining the variance in protein measurement reproducibility. We assessed both the aggregated ranks and the reproducibility measured in each individual study. We converted the protein reproducibility measurements from the three studies with replicates (ovarian, colon, CCLE) to ranks.

The potential factors such as protein abundance and unique peptides had a large range, therefore both the factors were log transformed and linear regression was performed.

Assessing the impact of protein abundance, protein variance, unique peptides, protein half-lives on the reproducibility of proteins (Figure S5D)

To understand the variance in protein reproducibility ranks explained by the potential factors (protein abundance, protein variance, unique peptides, and protein half-lives), we used four different linear models given by the equations -

- Protein abundance only: $rank(g) = \alpha + \beta * p_{abundance}(g)$
- Protein variance only: $rank(g) = \alpha + \beta * p_{variance}(g)$
- Unique peptides only: $rank(g) = \alpha + \beta * p_{peptides}(g)$
- Protein half-lives encoded as long and short: $rank(g) = \alpha + \beta_1 * p_{half-lives-long}(g) + \beta_2 * p_{half-lives-short}(g)$

CellPress
OPEN ACCESS

- Protein abundance, unique peptides and protein half-lives combined: $rank\ (g) = \alpha + \beta_1 * p_{abundance}(g) + \beta_2 * p_{variance}(g) + \beta_3 * p_{peptides}(g) + \beta_4 * p_{half-lives-long}(g) + \beta_5 * p_{half-lives-short}(g)$

where $rank\ (g)$ is the protein reproducibility rank for each protein, $p_{abundance}(g)$ is the protein abundance for each protein obtained from the GTEx project, $p_{variance}(g)$ is the variance of the protein abundance for each protein obtained from the GTEx project, $p_{peptides}(g)$ is the number of unique peptides for each protein obtained from the GTEx project,$p_{half-lives-long}(g)$ and $p_{half-lives-short}(g)$ are the half-lives of each protein (long and short) and the coefficients $\alpha,\ \beta,\ \beta_1,\ \beta_2,\ \beta_3,\ \beta_4$ and β_5 are computed using the ordinary least squares regression method. For all the models, protein reproducibility is assessed over the same set of proteins in each study. R^2 is used to assess the predictive power of the explanatory variables in explaining the variation of the response variable.

Pathway enrichment analysis

Pathway enrichment analysis was performed using the Mann-Whitney U test. Firstly, the KEGG pathways (Kanehisa et al., 2021) and their associated genes for *Homo sapiens* were downloaded using the KEGG API (https://www.kegg.jp/kegg/rest/keggapi.html). Only KEGG pathways with more than 3 genes with measured correlations were included for the enrichment analysis. The computed mRNA-protein correlations of CCLE and ovarian cancer studies were used to rank the proteins. A Mann-Whitney U test was performed to assess the rank of each pathway in each dataset and p-values obtained were corrected for false discovery rate (FDR) using the Benjamini-Hochberg method. For the figures presented in Figure 7 and S7 we specifically included pathways which have been previously identified as enriched in different cancer studies (Clark et al., 2019; Huang et al., 2021; Mertins et al., 2016; Zhang et al., 2014, 2016). To identify enriched pathways *after* accounting for experimental reproducibility, we regressed the CCLE and ovarian mRNA-protein correlation on both aggregated protein reproducibility ranks and mRNA-mRNA reproducibility correlations, which are based on the equations $c\ (g) = \alpha + \beta_1 * m\ (g) + \beta_2 * p\ (g)$, where $c\ (g)$ is the mRNA-protein correlation, $m\ (g)$ is the mRNA-mRNA reproducibility and $p\ (g)$ is the protein-protein reproducibility and the coefficients α, β_1 and β_2 are computed based on the ordinary least squares regression method. The residuals obtained from the regression were used to rank the proteins in pathway enrichment analysis. The top level categories (e.g., Metabolism, Genetic Information Processing) of the pathways were obtained from KEGG and are used to annotate the pathways in Figure 7 and S7.

QUANTIFICATION AND STATISTICAL ANALYSIS

Statistical analysis is described in the Method details and was carried out using Python 3.8, Pandas 1.2.5 (McKinney, 2011), numpy 1.20.2 (Harris et al., 2020), SciPy 1.7.1 (Virtanen et al., 2020) and StatsModels 0.12.2 (Seabold and Perktold, 2010). The figures were created with Matplotlib 3.3.4 (Hunter, 2007) and Seaborn 0.11.1 (Waskom, 2021).

Cell Reports Methods, Volume *2*

Supplemental information

Experimental reproducibility limits the correlation between mRNA and protein abundances in tumor proteomic profiles

Swathi Ramachandra Upadhya and Colm J. Ryan

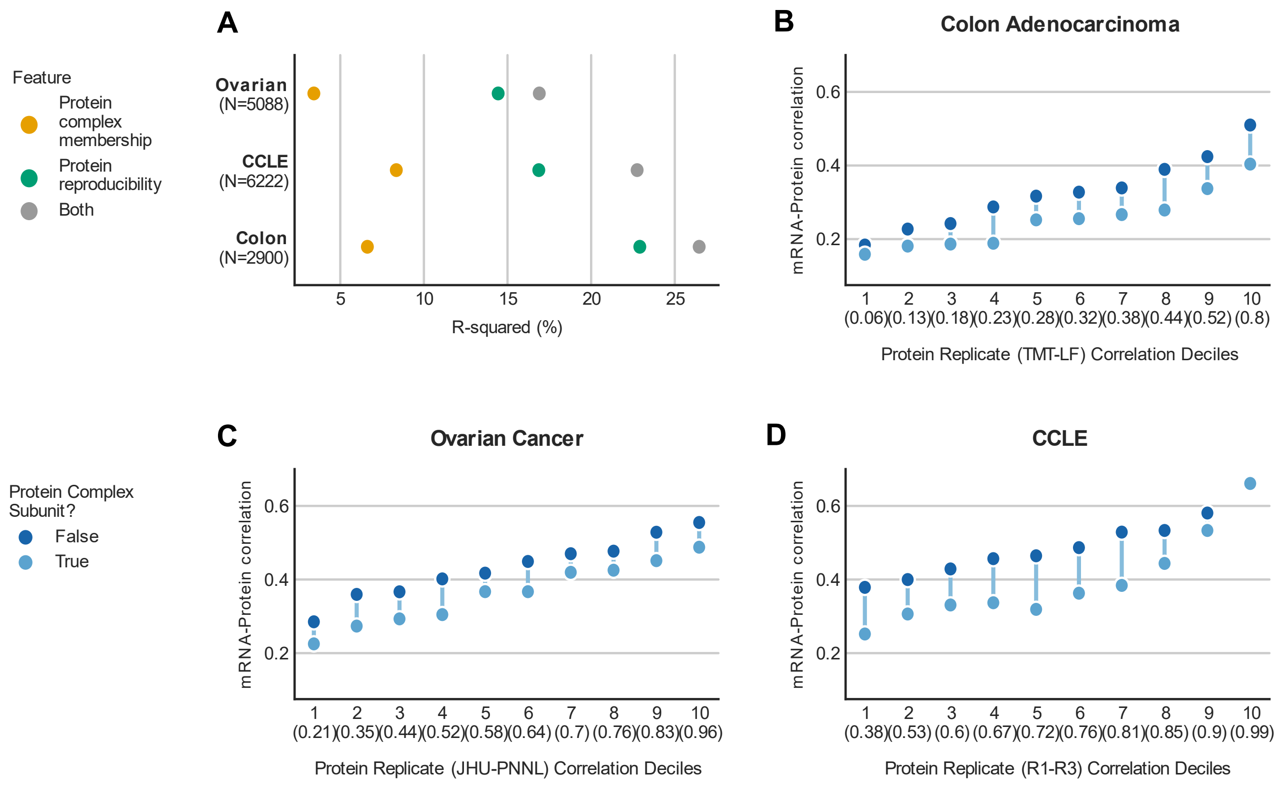

Figure S1. Protein complex membership and protein reproducibility contribute to the variation in mRNA-protein correlation. Related to Figure 2.

(A) Dot plot displaying the R-squared obtained from regressing mRNA-protein correlation of the indicated studies on protein complex membership and their corresponding protein reproducibility over the same set of proteins. The number of proteins considered for each analysis is specified in parentheses below the study on Y-axis. (B-D) Ranged dot plots showing the mean of mRNA-protein correlation for proteins that are complex subunits (light blue dot) or not (dark blue dot) within every decile of the proteomic replicates' correlation for colon (B) and ovarian tumour (C) and CCLE studies (D). The line represents the difference in the mean of the mRNA-protein correlation between the groups of proteins belonging to the same decile. X-axis indicates the decile number and contains the maximum correlation between the experimental proteomic replicates for that decile in parentheses.

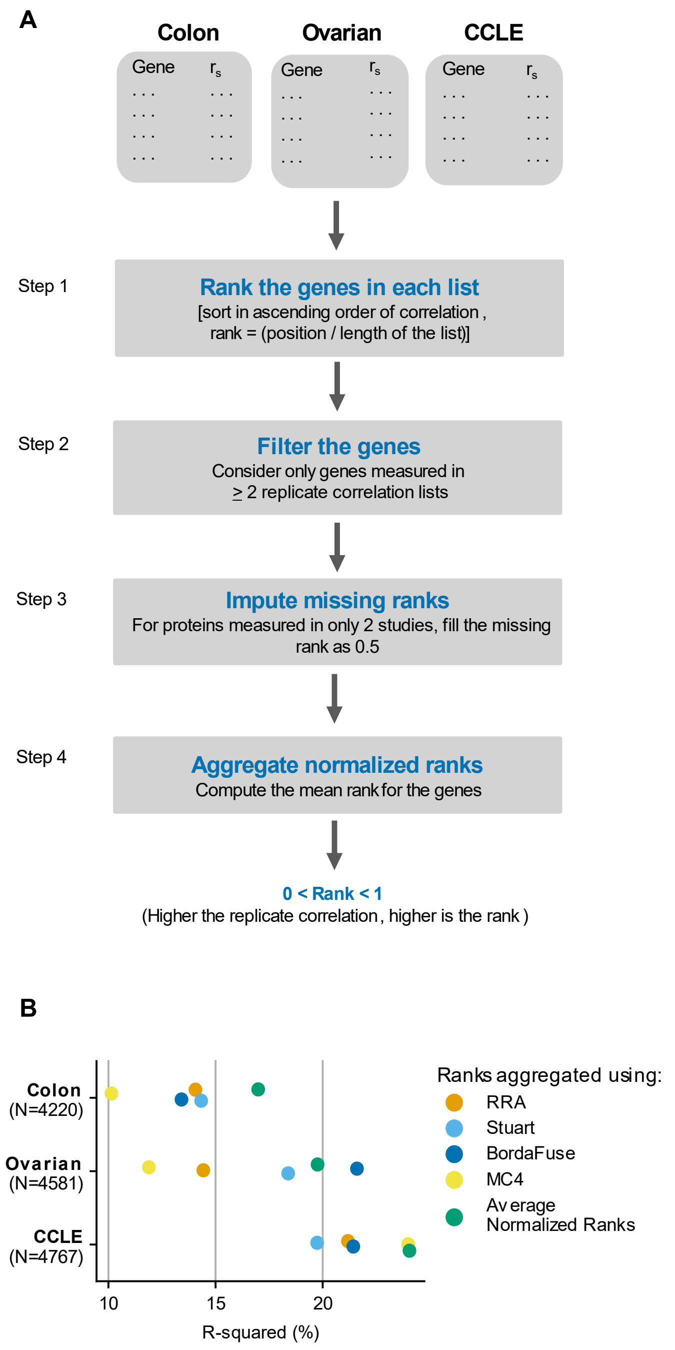

Figure S2. Aggregate protein reproducibility. Related to Figure 4.

(A) Workflow of our computational approach to aggregate the ranks of the correlation of experimental proteomic replicates from 3 different datasets - colon, ovarian and CCLE. The computed ranks lie between zero and one. The higher the correlation between the experimental proteomic replicates, the higher the rank. (B) Dot plot displaying R-squared obtained from regressing mRNA-protein correlation of the indicated studies on the aggregated protein ranks obtained from different algorithms (robust rank aggregation, Stuart, BordaFuse, Markov chain aggregator and our method of average normalized rank) over the same set of proteins. The number of proteins considered for each analysis is specified in parentheses below the study on Y-axis.

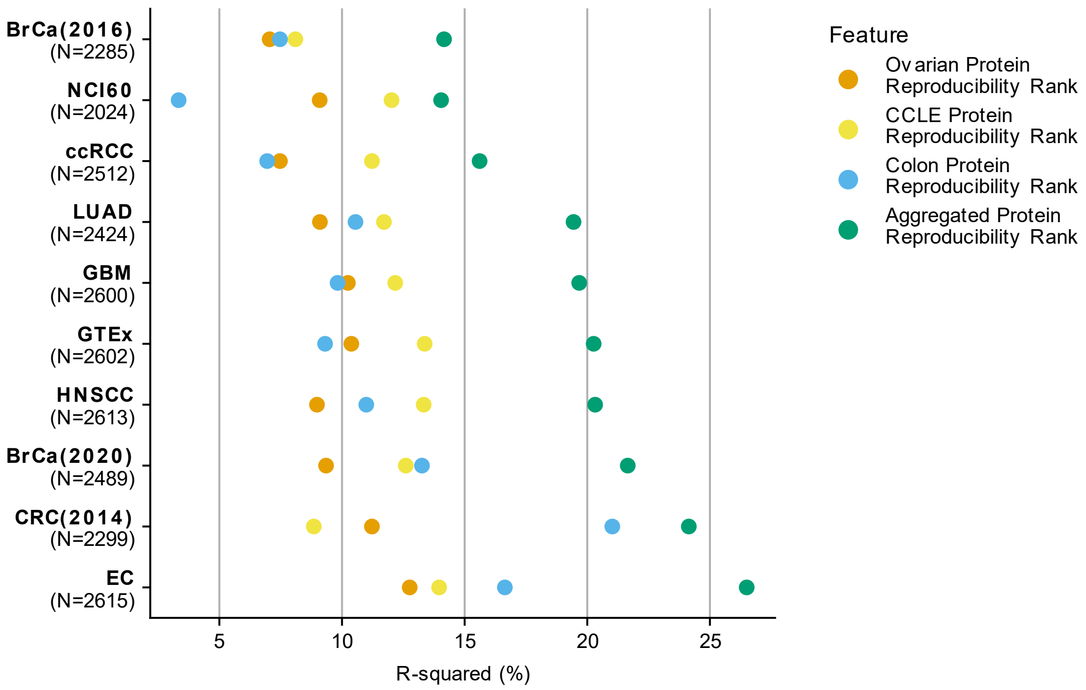

Figure S3. Aggregate protein reproducibility outperforms the individual protein reproducibility ranks in explaining the variation in mRNA-protein correlation. Related to Figure 4.

Dot plot comparing the R-squared values obtained from regressing mRNA-protein correlation on the individual protein reproducibility ranks and the aggregated protein reproducibility rank over the same set of proteins. The number of proteins considered for each analysis is specified in parentheses below the study on Y-axis.

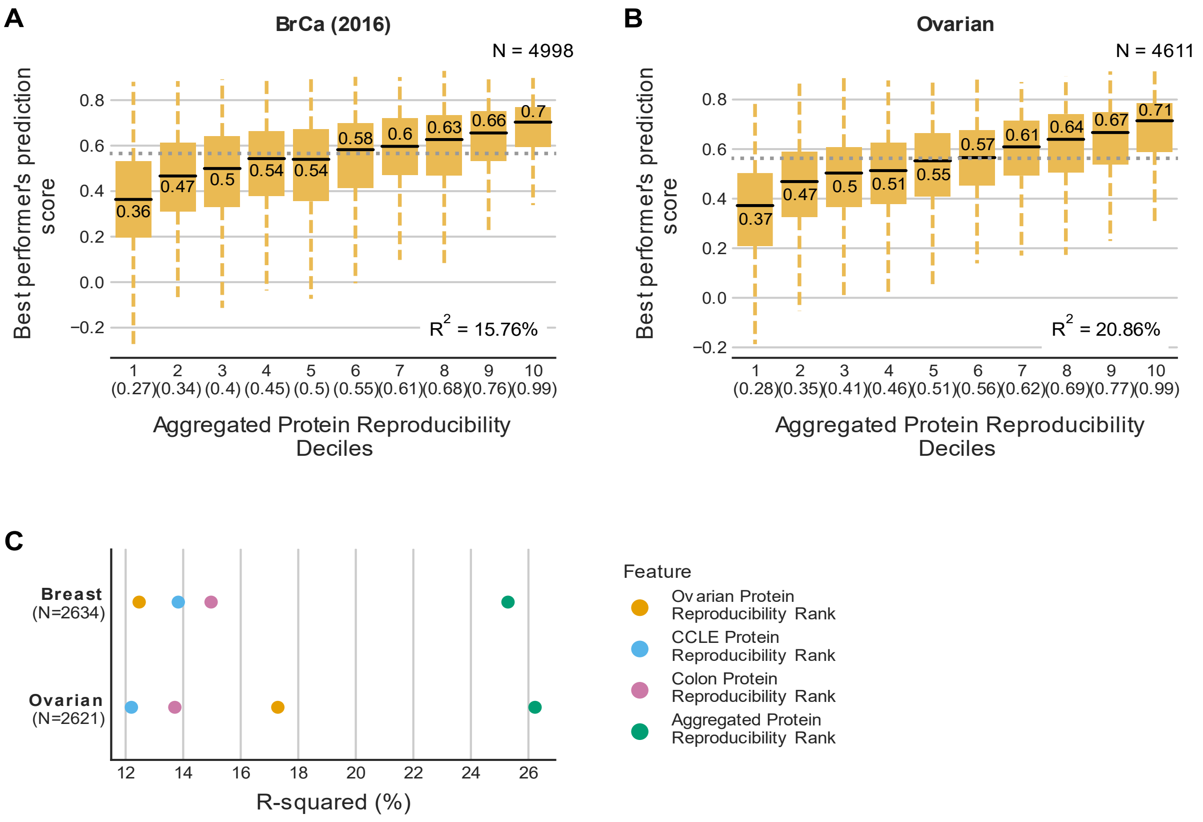

Figure S4. Proteins that are highly reproducible can be better predicted using machine learning. Related to Figure 4.

Boxplots showing the distribution of prediction scores from the best performing model in the NCI CPTAC DREAM Proteogenomics challenge for proteins in each decile of the aggregated protein reproducibility ranks in breast (A) and ovarian studies (B). The prediction score is the Pearson correlation between the observed and predicted protein abundance. The decile is indicated on the X-axis along with the highest score of the aggregated protein reproducibility rank present within that decile. For each box plot, the black central line represents the median, the top and bottom lines represent the 1st and 3rd quartile, and the whiskers extend to 1.5 times the interquartile range past the box. Outliers are not shown. The median of each decile is indicated above/below the black central line for each box plot. The overall median of the prediction score from the best performing model is indicated as a dotted gray line in each plot. The R^2 obtained from regressing the prediction score on the aggregated protein reproducibility ranks is in the bottom-right corner. (C) Dot plot comparing the R-squared values obtained from regressing protein abundance prediction scores of breast and ovarian tumour studies, obtained from NCI CPTAC Proteogenomics DREAM Challenge, on the individual protein reproducibility ranks and the aggregated protein reproducibility rank over the same set of proteins. The number of proteins considered for each analysis is specified in parentheses below the study on Y-axis.

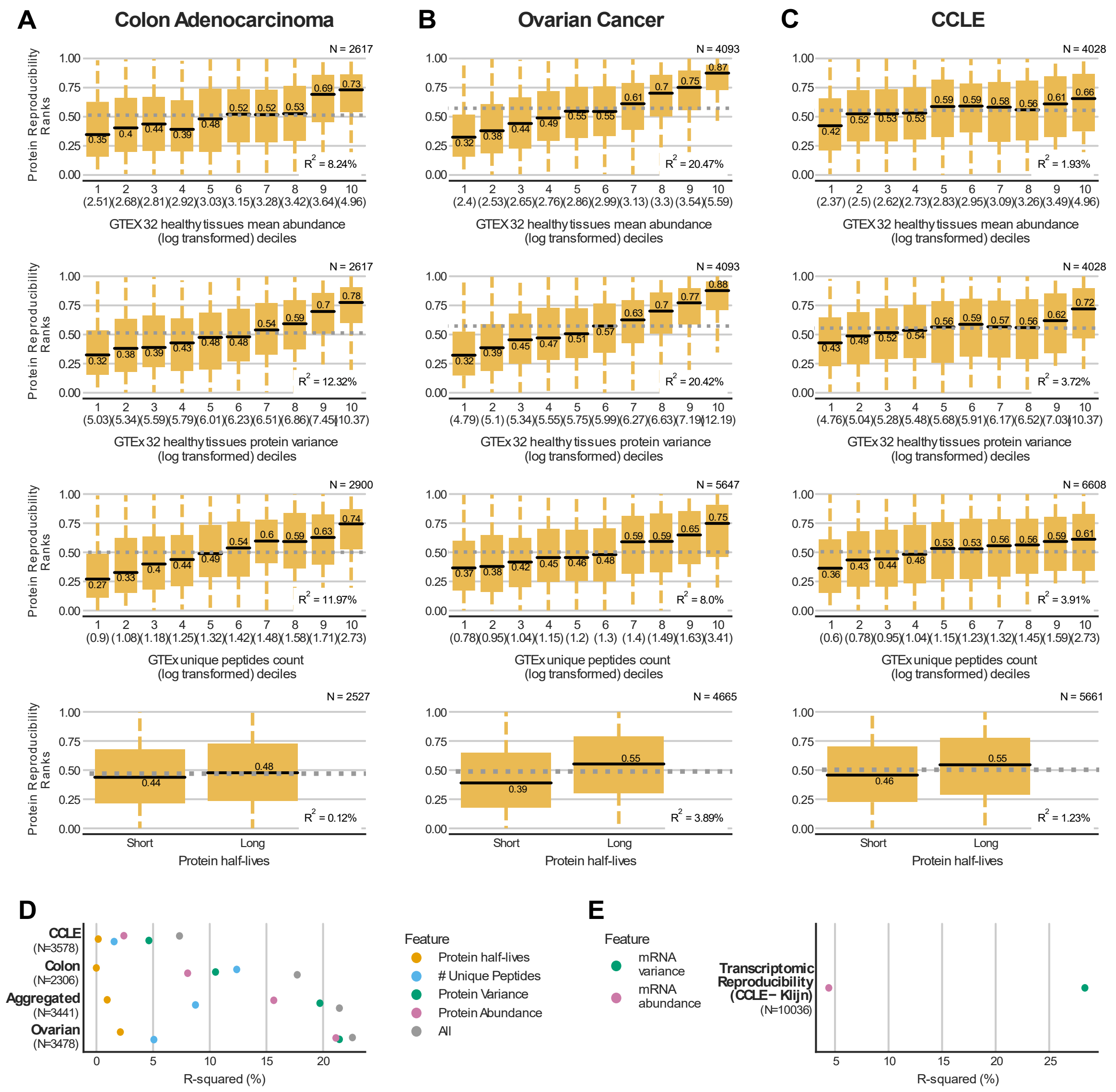

Figure S5. Potential factors influencing protein reproducibility (in individual studies with experimental proteomic replicates) and transcriptomic reproducibility. Related to Figure 5 and 6.

(A-C) Similar to Fig. 5 but for the individual protein reproducibility ranks from each study. (D) Dot plot comparing the R-squared values obtained from regressing the individual protein reproducibility ranks and the aggregated protein reproducibility ranks on the potential factors that affect protein reproducibility - protein abundance, variance of protein abundance, unique peptides, protein half-lives individually and all of them collectively over the same set of proteins. (E) Dot plot comparing the R-squared values obtained from regressing the potential factors that affect transcriptomic reproducibility – mRNA abundance and mRNA variance. The number of proteins considered for each analysis is specified in parentheses below the study on Y-axis.

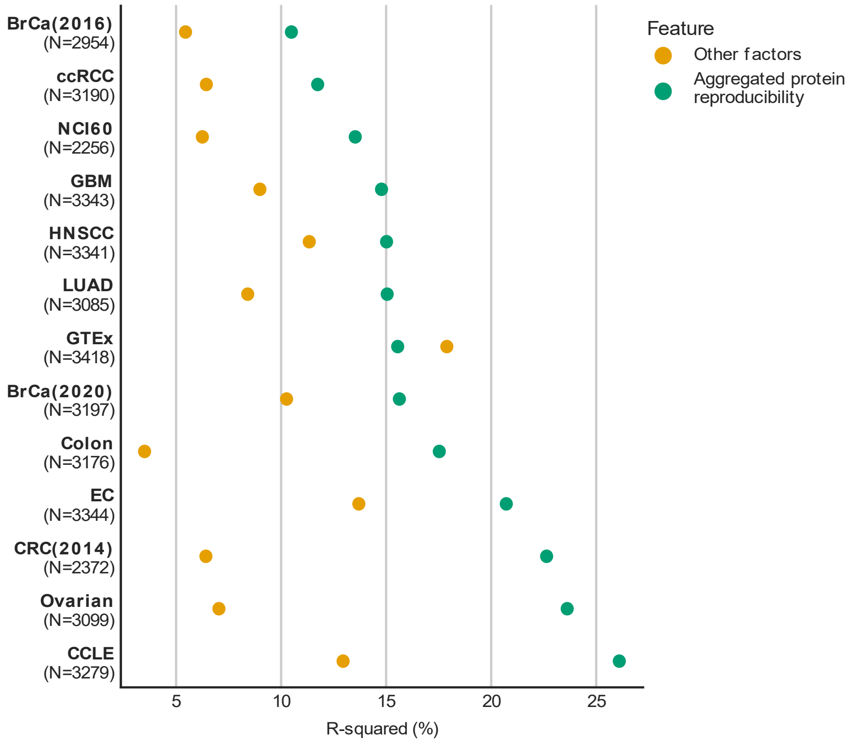

Figure S6. Protein reproducibility explains the variation in mRNA-protein correlation better than the other factors (protein abundance, protein variance, unique peptides, protein half-life). Related to Figure 5.

Dot plot comparing the R-squared values obtained from regressing mRNA-protein correlation of the studies on the aggregated protein reproducibility ranks and the potential factors that affect protein reproducibility - protein abundance, unique peptides, protein half-lives individually over the same set of proteins. The number of proteins considered for each analysis is specified in parentheses below the study on Y-axis.

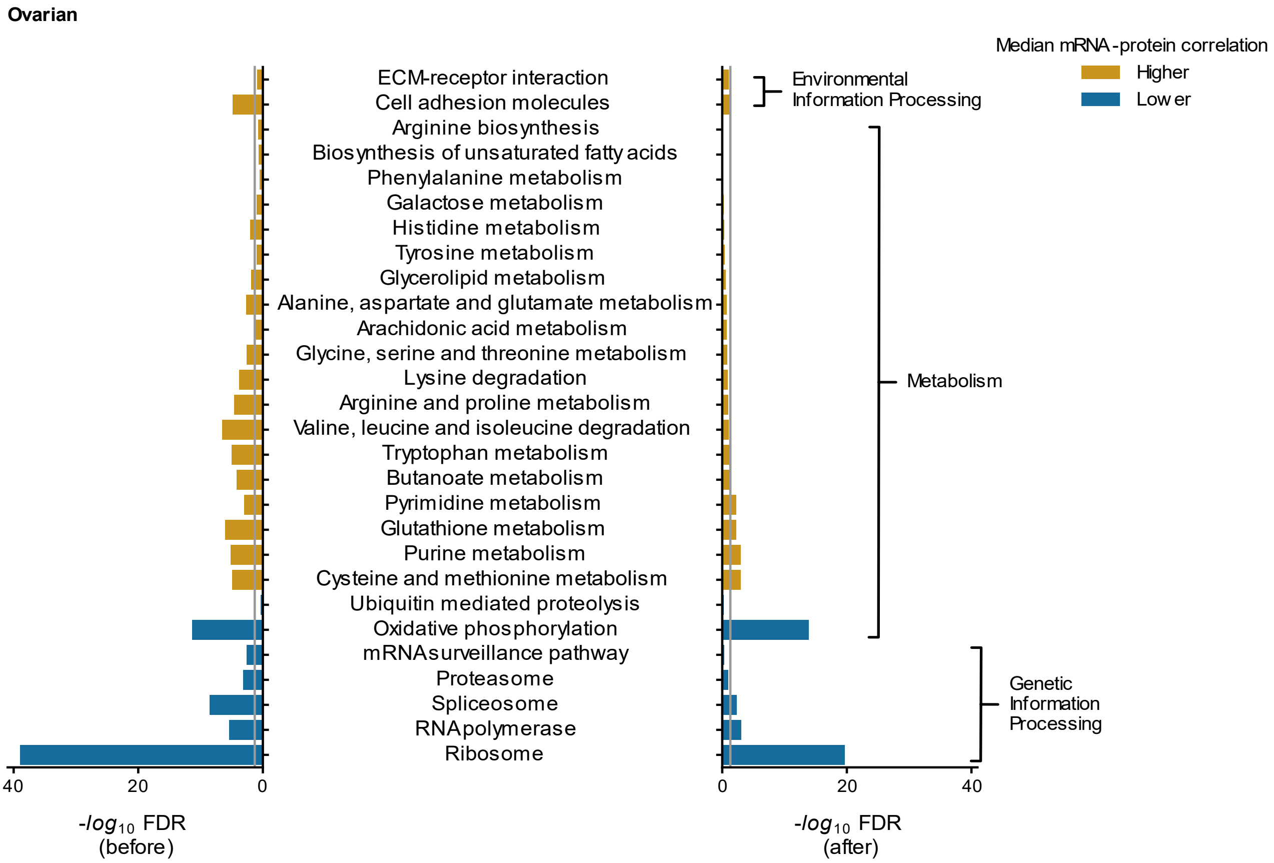

Figure S7. KEGG pathways enrichment analysis for ovarian cancer study. Related to Figure 7. Bar charts displaying the KEGG pathway enrichment analysis of the ovarian cancer study mRNA-protein correlation before (left) and after (right) accounting for protein-protein and mRNA-mRNA reproducibility. The $-\log_{10}$ of Benjamini-Hochberg FDR corrected p-values calculated using Mann-Whitney U test is deemed as enrichment for the pathway. For each bar chart, the gray line indicates the threshold considered for significant enrichment (FDR < 0.05). If the enrichment is below the threshold, then it is not considered significant. The bars are coloured orange if the median mRNA-protein correlation of genes within the pathway > median mRNA-protein correlation of genes *not* in the pathway, otherwise the bars are coloured blue.
